# TRPA1 for Butterfly Eyespot Formation

**DOI:** 10.3390/ijms27031420

**Published:** 2026-01-30

**Authors:** Momo Ozaki, Joji M. Otaki

**Affiliations:** The BCPH Unit of Molecular Physiology, Department of Chemistry, Biology and Marine Science, Faculty of Science, University of the Ryukyus, Nishihara, Okinawa 903-0213, Japan

**Keywords:** butterfly wing, color pattern formation, eyespot, organizer, TRPA1

## Abstract

Butterfly wing color pattern formation is a process of two-dimensional morphogenesis involving long-range lateral signaling in pupal wing tissues. We hypothesized that TRP (transient receptor potential) channels, which are multimodal sensors for various stimuli, are involved in this developmental process. Using the blue pansy butterfly *Junonia orithya*, we injected the TRPA1 antagonists, AM0902 and AP-18, and an agonist, JT010, into pupae and observed that the eyespot core disk area in adult wings increased and decreased in response to AM0902 and JT010, respectively, although AP-18 did not induce any change. Furthermore, the eyespot outer black ring area increased in response to AM0902, and the orange ring area increased in response to JT010. We detected TRPA1 mRNA via RT-PCR in the pupal wing tissues of this species. An antibody against the *J. orithya* TRPA1 extracellular site induced unique aberrant color patterns with wing vein defects. These results suggest that TRPA1 is expressed in pupal wing tissue and may integrate signaling information to determine eyespot size and structure in butterfly wings. TRPA1 likely suppresses the black core disk and the outer black ring and enhances the nonblack orange ring in eyespots during development.

## 1. Introduction

One of the important discoveries in sensory physiology in recent decades is the family of transient receptor potential (TRP) channels, which are multimodal ion channels that can be activated by various noxious stimuli, including high and low temperatures, chemical substances (including plant secondary metabolites), and mechanical stresses [1]. The *trp* gene was originally cloned on the basis of the blind behavioral phenotype of *Drosophila* during prolonged intense light [2,3,4] and was shown to be a light-activated cation channel [2,5]. On the other hand, a mammalian capsaicin receptor gene was independently cloned via a cell culture system expressing murine cDNA; the gene was found to encode a novel TRP channel that is also activated by heat [6,7,8]. Since then, various TRP subfamilies have been identified widely in the animal kingdom [9,10]. Among them, diverse TRPA (ankyrin) genes have been identified in insects [9,10], and most insects appear to have four or five TRPA subfamily members [9]. For example, *Drosophila melanogaster* has four TRPA genes, including *trpa1* and *painless* (*pain*) [9].

TRPA1 is required for sensing temperatures in *Drosophila* [11,12,13]. However, its function is rather broad, reflecting its multimodality. For example, TRPA1 in *Drosophila* functions as a gustatory receptor [11,14,15,16]. TRPA1 is also employed as a gustatory receptor that integrates temperature and chemical information in the moth *Maduca sexta* [17]. Similarly, olfactory chemical nociception is mediated by TRPA1 in various insects [18,19,20,21]. Certain TRPA1 channels can detect hydrogen peroxide generated via ultraviolet light [22,23]. Likewise, heat and physical damage may also induce hydrogen peroxide and other reactive oxygen species (ROS), which are detected by TRPA1 [24]. Because TRPA1 is multimodal, cell types in which TRPA1 is expressed are crucial for its function as either a thermal or chemical sensor [25]. In addition to thermal nociception [26], mechanical nociception is mediated by TRPA1, which senses shear stress [27,28]. Similar functions have been ascribed to Painless (Pain), another TRPA family member in *Drosophila* that functions as a noxious heat sensor [29,30,31] and a noxious chemical sensor [32]. In the tomato leafminer moth *Tuta absoluta*, Painless functions in temperature preference [33]. Moreover, Painless plays a crucial role in sexual behavior in *Drosophila* [34,35,36].

In addition to these neuronal functions for sensing environmental stimuli, TRPA1 appears to regulate several developmental events. For example, TRPA1 regulates actomyosin-generated forces to complete dorsal closure of cellular sheets during embryonic development in *Drosophila* [37]. Additionally, in *Drosophila*, intestinal stem cell proliferation is mediated by TRPA1, which is activated by shear stress [28]. TRPA1 is involved in transgenerational diapause determination during embryonic development in silkworms [38,39]. In these cases, TRPA1 seems to be expressed in nonexcitable cells, which contributes to the autonomous (nonneuronal) regulation of tissues during development. However, the current knowledge concerning the developmental functions of TRPA1 is still limited. To understand the developmental functions of TRPA1, studies on various developmental systems may be encouraged.

We have been studying the developmental mechanisms of color pattern formation in butterfly wings [40]. Butterfly wing color patterns are determined during the early pupal stage in wing epidermal (epithelial) cells, which form a monolayer cellular sheet [41,42,43,44,45]. Butterfly wing color patterns are often diversely expressed in response to larval environmental conditions such as light, temperature, and humidity, even within a single species. This type of phenotypic plasticity is well known in the genus *Junonia* (Lepidoptera, Nymphalidae) [42,46,47,48,49,50]. One good example is probably the blue pansy butterfly *Junonia orithya* [49,50]. This species is sexually dimorphic and exhibits seasonal morphs under natural conditions, likely in response to temperature and light conditions [50]. In addition, as an experimental system, a characteristic color pattern can be induced by temperature shock [49,51] and pharmacological treatments [52,53] in this and related species. A chitin-binding chemical compound called fluorescent brightener 28 (FB28) can induce a color pattern that is similar, if not identical, to a color pattern induced by cold shock [54]. Other unrelated chemical compounds, such as tungstate [55], acid carboxypeptidase [56], and sulfated polysaccharides [57], can produce similar color patterns. Temperature-induced color patterns are also regulated by a cold shock hormone, the identity of which appears to be dopamine or other related biogenic amines [58]. Stress inducers such as thapsigargin, geldanamycin, and ionomycin induce a dark color pattern with less clear elemental boundaries [52]. Ecdysteroids make the background brighter [52]. To form a proper eyespot pattern, pupal wing tissues require a physical surface with proper rigidity and hydrophobicity [59]. Recently, we demonstrated that pharmacological inhibition of the mechanical receptor PIEZO1 results in an increase in eyespot size in *J. orithya*, suggesting that mechanical signals play a role in eyespot color pattern determination in butterflies [60]. The possible contribution of mechanical signals that may be generated by cellular enlargement or duplication in conjunction with cellular binding to the cuticle has been proposed as the physical distortion hypothesis [40] or the cuticle hypothesis [45]. The results of these experiments suggest that physical and chemical information should be integrated to finalize developmental fate determination in pupal wing tissues.

**Figure 1:**
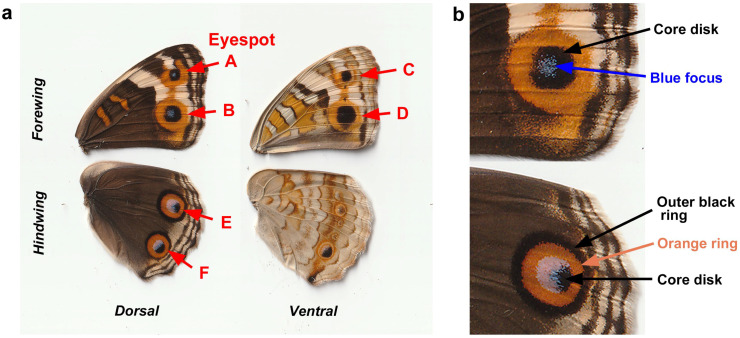
Female wing color patterns of the blue pansy butterfly *J. orithya*. (**a**) Dorsal and ventral wings. Eyespots A–F are indicated. (**b**) Subelements of the dorsal forewing and hindwing eyespots (Eyespots B and E).

Here, we investigated the possible developmental functions of TRPA1 in butterfly wing color pattern formation. Because developmental fate must be determined by the integration of information from several transduction pathways with various modalities, we reasoned that TRPA1 is a reasonable candidate for information integration for developmental fate determination in wing epidermal cells. In the present study, we pharmacologically treated fresh pupae with chemical modulators of TRPA1: AM0902 (antagonist) [61,62], AP-18 (antagonist) [63,64,65,66], and JT010 (agonist) [67,68,69,70,71], which were all dissolved in dimethyl sulfoxide (DMSO). We then observed changes in the eyespot color pattern of adult wings after eclosion. Using the blue pansy butterfly *J. orithya*, which has many eyespots on the wing surface (Figure 1a), we focused on the dorsal forewing eyespots (Eyespots A and B), ventral forewing eyespots (Eyespots C and D) and dorsal hindwing eyespots (Eyespots E and F) of females (Figure 1a). We quantitatively evaluated size changes in these whole eyespots and their subelements (Figure 1b). Because DMSO is known to induce a significant increase in eyespot size [60], we also included a DMSO treatment group for comparison. If that was not possible due to the number of individuals available in a sibling group, we numerically corrected the untreated group for the DMSO treatment [60]. We detected TRPA1 mRNA via RT-PCR (reverse transcriptase-polymerase chain reaction), from which we obtained cDNA nucleotide sequences. Protein amino acid sequences that were obtained after the conceptual translation of cDNA nucleotide sequences were used to design peptide epitopes for polyclonal antibodies, which were then used for functional analysis.

## 2. Results

### 2.1. TRPA1 Modulators on Eyespot Core Disks

**Figure 2:**
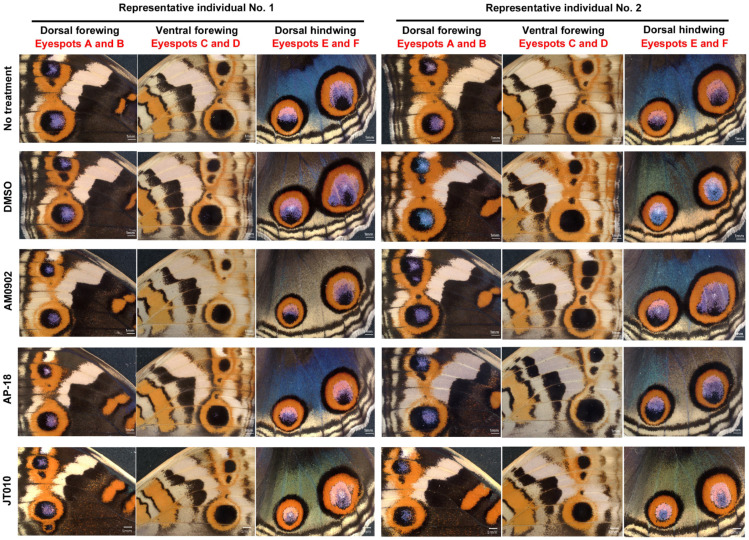
Representative wing color patterns of blue pansy butterflies after treatment with TRPA1 chemical modulators. Two individuals (No. 1 and No. 2) are shown in each treatment. Wing images of all individuals used in this study are shown in Supplementary Figures S1–S5.

We first examined the effects of AM0902 (TRPA1 antagonist; *n* = 11; *n* indicates the number of females with successful eclosion), AP-18 (TRPA1 antagonist; *n* = 9), and JT010 (TRPA1 agonist; *n* = 13). By qualitative visual inspection, we did not observe any changes in the overall color patterns on any wing surfaces (dorsal or ventral sides of the forewing or hindwing) compared with those in the DMSO-treated group (*n* = 18 for the sibling groups treated with AM0902 and AP-18; *n* = 11 for the sibling group treated with JT010) (Figure 2).

**Figure 3:**
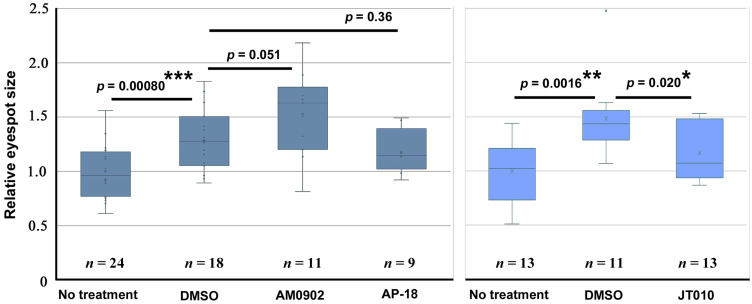
Quantitative evaluation of the area of eyespot black core disks on the ventral forewing after treatment with TRPA1 modulators. Two panels (**left**, **right**) indicate two trials with different sibling groups. Asterisks indicate statistical significance (*: *p* < 0.05, **: *p* < 0.01, ***: *p* < 0.001).

For the quantitative evaluation of the black core disk area of all eyespots on the ventral forewing, we first confirmed that, compared with the no treatment group, DMSO treatment (*n* = 19) significantly increased the area value (*n* = 24) (*p* = 0.00080) in the first sibling group (Figure 3, left). A similar result was obtained in another sibling group treated with DMSO (*n* = 11) compared with the no treatment group (*n* = 13) (*p* = 0.0016) (Figure 3, right). Hence, other treatments were compared with the DMSO treatment group of the same sibling group because all the chemical compounds used in this study were dissolved in DMSO.

AM0902 resulted in an enlargement of the core disk area on the ventral forewing (*n* = 11) compared with the DMSO treatment group (*n* = 18) (Figure 3, left). Although its *p*-value was just above the conventional significance level (*p* = 0.051), we considered this case to be pharmacologically important, considering that it was scored beyond the DMSO-induced enlargement and that the effects of DMSO and AM0902 may not be linearly additive. In contrast, AP-18 (*n* = 9) did not induce any change (*p* = 0.36) (Figure 3, left), indicating no effect of AP-18 and suggesting a specific effect of AM0902. On the other hand, in the different sibling group, the JT010 treatment group (*n* = 13) showed significantly lower area values than the DMSO treatment group (*n* = 11) (*p* = 0.020) (Figure 3, right).

### 2.2. TRPA1 Modulators on Various Eyespots

**Figure 4:**
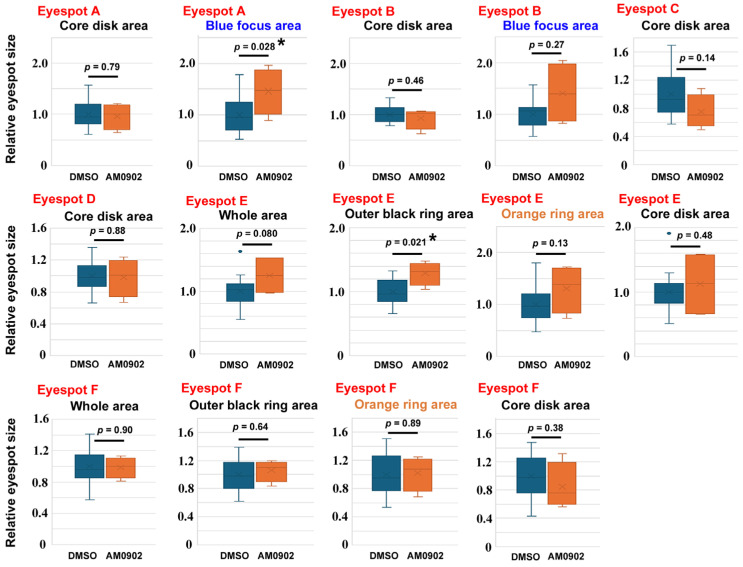
Effect of AM0902 on eyespot size. For Eyespots A–F, refer to Figure 1. Among these 14 areas of measurement, the blue focus area of Eyespot A and the outer black ring area of Eyespot E are statistically significant. Asterisks indicate statistical significance (*: *p* < 0.05).

**Figure 5:**
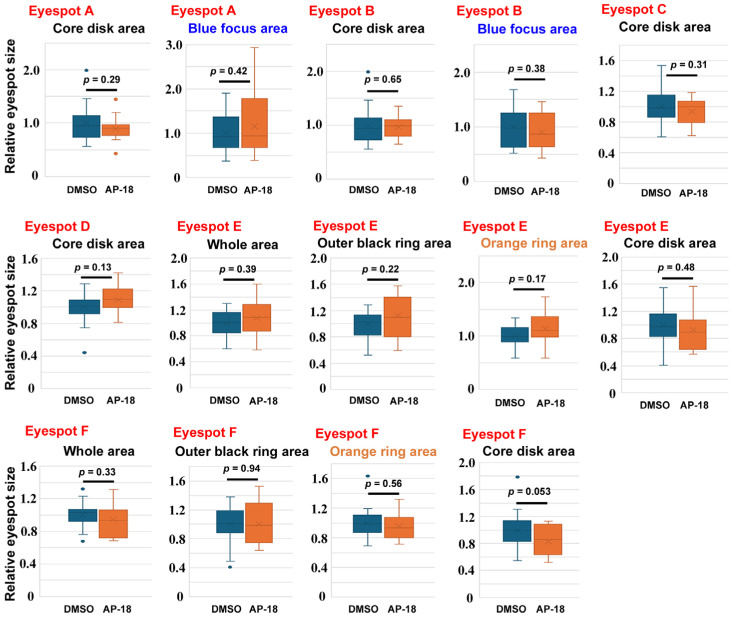
Effect of AP-18 on eyespot size. For Eyespots A–F, refer to Figure 1. Among these 14 areas of measurement, no area is statistically significant.

**Figure 6:**
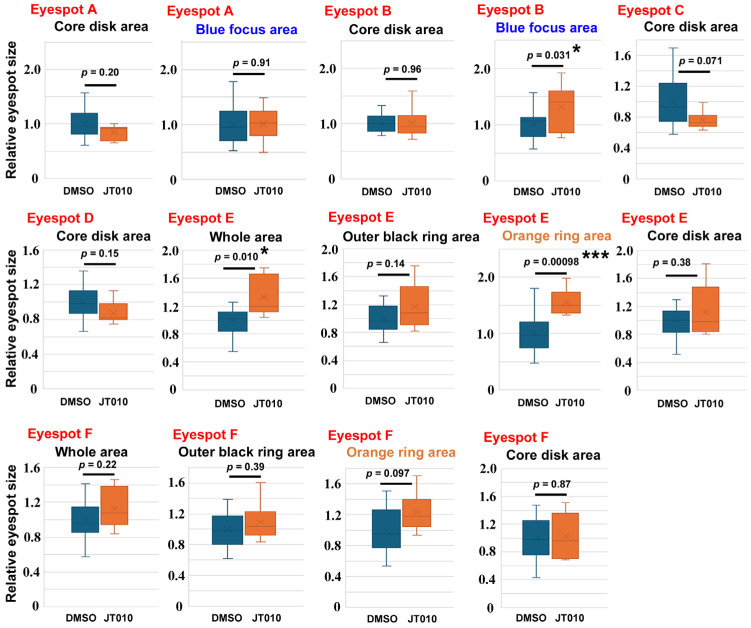
Effect of JT010 on eyespot size. For Eyespots A–F, refer to Figure 1. Among these 14 areas of measurement, the blue focus area of Eyespot B, the whole area of Eyespot E, and the orange ring area of Eyespot E are statistically significant. Asterisks indicate statistical significance (*: *p* < 0.05, ***: *p* < 0.001).

To confirm these findings, we examined the effects of AM0902 (*n* = 4) (Figure 4), AP-18 (*n* = 15) (Figure 5), and JT010 (*n* = 6) (Figure 6) in different sibling groups. Here, 14 area traits associated with six eyespots (Eyespots A–F) were measured and compared individually. In the case of AM0902, the blue focus area in Eyespot A (*p* = 0.028) and the outer black ring area of Eyespot E (*p* = 0.021) significantly increased (Figure 4), confirming the increasing trend of eyespot areas observed in the previous section. In the case of AP-18, no significant change was observed (Figure 5). In the case of JT010, the orange ring area (*p* = 0.00098) and the whole area of Eyespot E (*p* = 0.010) significantly increased, although the black areas did not decrease significantly (Figure 6). Additionally, the blue focus area of Eyespot B significantly increased (*p* = 0.0031) (Figure 6).

### 2.3. RT-PCR for TRPA1

**Figure 7:**
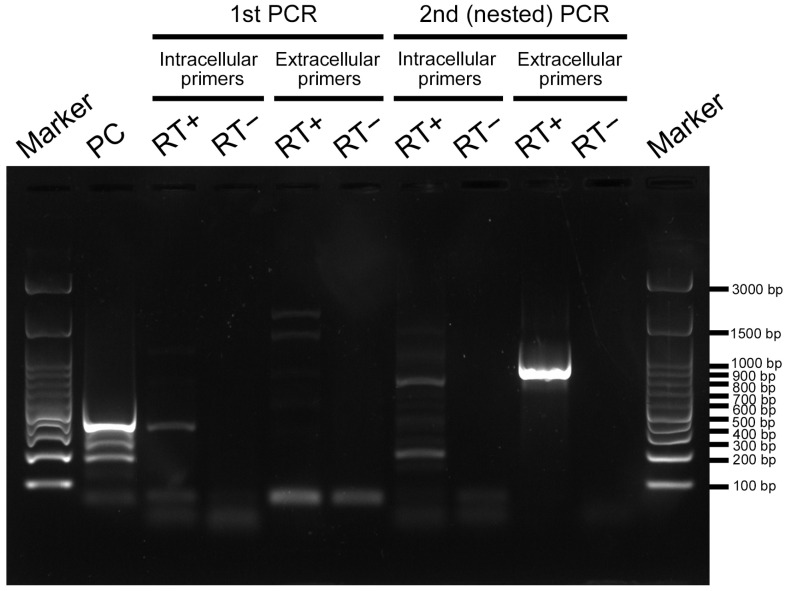
RT-PCR for TRPA1 from pupal wing tissues of *J. orithya*. The original gel image is shown in Supplementary Figure S6.

To examine whether TRPA1 is expressed in pupal wing tissues during the critical period of color pattern determination, we performed RT-PCR using two primer sets designed from the TRPA1 gene of a related species, *Vanessa cardui*. One primer set corresponded to the intracellular site, and the other primer set corresponded to the extracellular site. After the second (nested) PCR, we detected the amplified PCR products of approximately 700–900 bp in length as expected, although an unexpected smaller product of 200–300 bp also appeared in the intracellular primer set (Figure 7). The PCR products of 700–900 bp were subsequently sequenced. The product with the intracellular primers was 730 bp in length (GenBank Accession Number: PX675203), excluding the primers used. The product with the extracellular primers was 835 bp in length (GenBank Accession Number: PX675202), excluding the primers used. They were confirmed to be partial sequences of TRPA1 from *J. orithya* via similarity search using BLAST (Basic Local Alignment Search Tool). Their amino acid sequences were obtained via conceptual translation, on the basis of which polyclonal antibodies against peptide epitopes from these two regions of *J. orithya* TRPA1 were produced.

### 2.4. Anti-TRPA1 Antibodies

**Figure 8:**
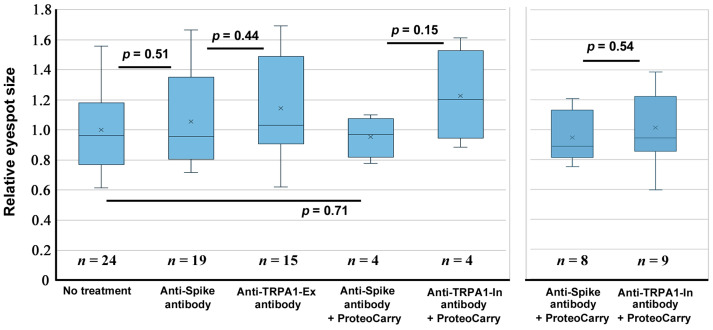
Effect of anti-TRPA1 antibodies on eyespot size. Two panels (**left**, **right**) indicate two trials with different sibling groups. None is statistically significant.

We examined the possible effects of anti-TRPA1 antibodies on eyespot size via injection. Qualitatively, we did not observe any noticeable change in the individuals treated with any of the antibodies used here. We then evaluated the eyespot black core element areas on the ventral forewings as described in Section 2.1. In the case of the antibody against an extracellular portion of TRPA1 (anti-TRPA1-Ex antibody) (*n* = 15), no significant change was observed in comparison to the anti-spike antibody group (*n* = 19) (*p* = 0.44) (Figure 8, left). Similarly, in the case of the antibody against an intracellular portion of TRPA1 (anti-TRPA1-In antibody) applied together with ProteoCarry (*n* = 4), we did not observe any statistically significant change in comparison to the anti-spike antibody group with ProteoCarry (*n* = 4) (*p* = 0.15) (Figure 8, left). We repeated the injections of the anti-TRPA1-In antibody in a different sibling group (*n* = 9), but we observed no statistically significant difference in comparison to the anti-spike antibody group (*n* = 8) (*p* = 0.54) (Figure 8, right).

**Figure 9:**
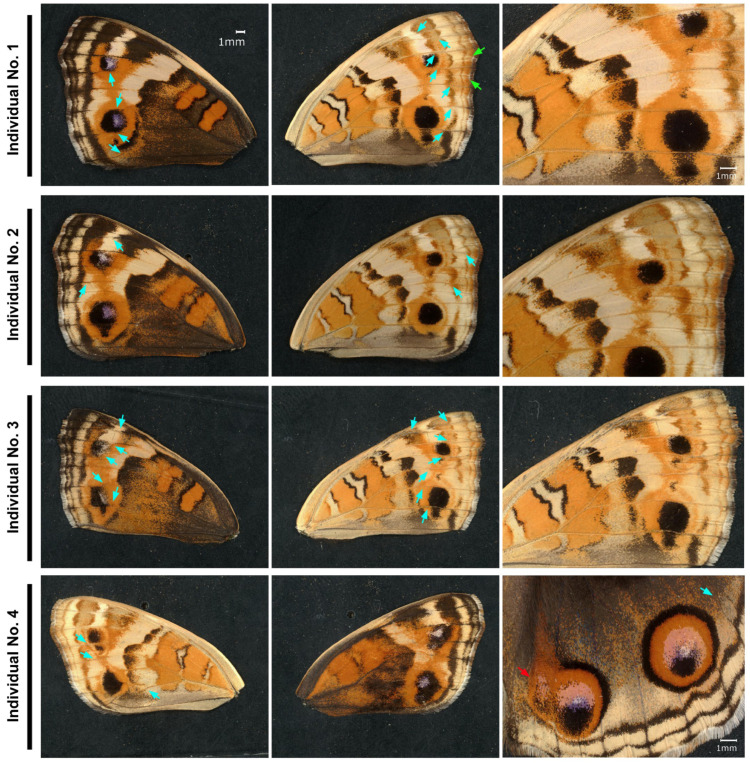
Effects of the anti-TRPA1-Ex antibody on eyespot shape, wing veins, and midlines. The light blue arrows indicate curved wing veins, and the light green arrows indicate doubled midlines. The red arrow indicates a deformed extra eyespot on the right dorsal hindwing.

Although we could not detect any change in eyespot size in the previous sibling groups, we injected the anti-TRPA1-Ex antibody into another sibling group (the second trial). Here, we observed clear eyespot shape changes in some individuals treated with the anti-TRPA1-Ex antibody (Figure 9). In these individuals, extensive orange expansion was observed on both the dorsal and ventral hindwing surfaces, and the shape of the eyespot black core disk was deformed to be triangular or square (Figure 9). A closer inspection revealed that these eyespot changes were associated with the aberration of wing veins and midlines. Among the treated individuals (*n* = 34; 14 males and 20 females), approximately one-quarter presented wing vein aberrations with various degrees of color pattern modifications (4 males and 5 females); the aberration rate was 26.5% in total. In these individuals, wing veins and midlines were not straight but tortuous, especially in the area where the eyespots were located (Figure 9). No aberrations were detected in the no treatment group (*n* = 18; 7 males and 11 females). In one individual, the posterior eyespot of the right dorsal hindwing was deformed, and in its adjacent wing compartment, a deformed extra eyespot emerged (Figure 9).

**Figure 10:**
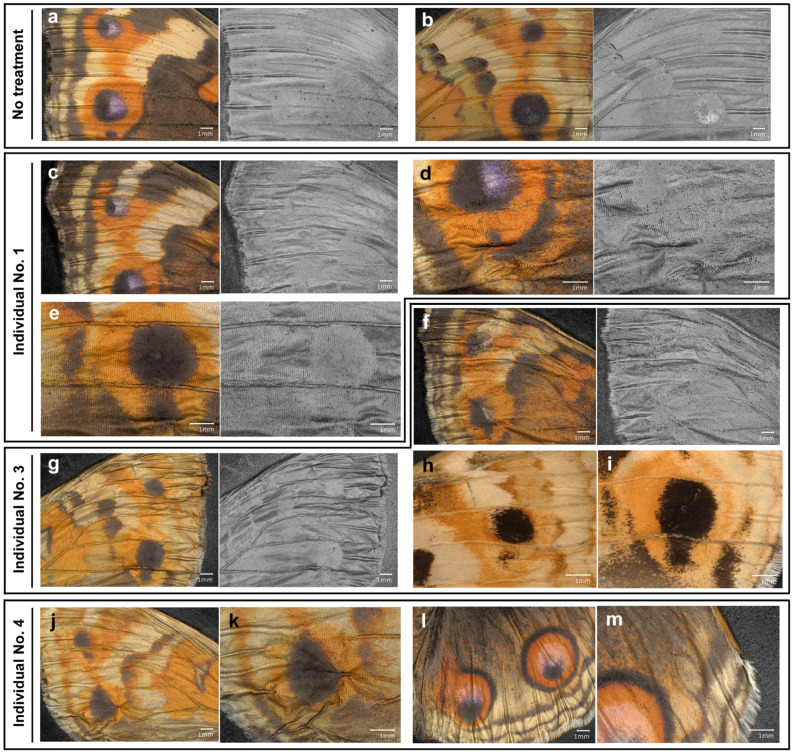
Deep contrast images of female individuals treated with the anti-TRPA1-Ex antibody. A pair of color and monochrome images in the same visual field is shown in (**a**–**g**). Individual numbers here correspond to those of Figure 9. (**a**,**b**) Forewing from an individual in the no treatment group for comparison. (**a**) Dorsal forewing. (**b**) Ventral forewing. (**c**–**e**) Forewing from an individual treated with the anti-TRPA1-Ex antibody (Individual No. 1). (**c**) Dorsal forewing. (**d**) Eyespot B of the wing shown in (**c**). (**e**) Eyespot D of the wing shown in (**c**). (**f**–**i**) Forewings from yet another individual treated with the anti-TRPA1-Ex antibody (Individual No. 3). (**f**) Dorsal forewing. (**g**) Ventral forewing. (**h**) Eyespot C of the wing shown in (**g**). (**i**) Eyespot D of the wing shown in (**g**). (**j**–**m**) Wings from additional individual treated with the anti-TRPA1-Ex antibody (Individual No. 4). (**j**) Ventral forewing. (**k**) Eyespot D of the wing shown in (**j**). (**l**) Dorsal hindwing. (**m**) Anterior portion of the dorsal wing shown in (**l**).

We further examined the wings of the individuals treated with the anti-TRPA1-Ex antibody using deep contrast images, which highlighted height information, making the visual identification of wing veins and midlines easier (Figure 10). In the forewing with no treatment, we observed normal wing veins, midlines, and wing surfaces both in the dorsal and ventral sides (Figure 10a,b). That is, wing veins and midlines were regularly positioned. Interestingly, there were “normal distortions” (i.e., height differences) on the wing surface associated with wing veins and midlines (Figure 10a,b). In contrast, in the forewings and hindwings treated with the anti-TRPA1-Ex antibody, wing veins and midlines were irregularly tortuous, and wing surfaces were not flat (Figure 10c–m). There were numerous irregular distortions of wing surfaces, especially around eyespots (Figure 10c–m).

## 3. Discussion

In this study, we investigated the possible involvement of TRPA1 in color pattern formation in butterfly wings. The involvement of TRPA1 may be reasonable based on the following considerations. First, calcium waves propagate from the prospective eyespot focus and damage site in the wing tissue during the critical period of color pattern determination [72,73], suggesting that cation channels may be involved in calcium wave production and propagation. Second, a mechanical surface is required for eyespot development [59,60]. TRP can be activated by a mechanical surface. Third, the color of a scale cell should be determined not only by a single input. Instead, it should be determined by multiple inputs including mechanical and chemical stimuli. TRP channels are multimodal, suggesting that they may integrate various signal inputs from different modalities in the critical period of color pattern determination in butterfly wings.

We executed a pharmacological approach in this study with AM0902, AP-18, and JT010, which is reasonable, considering that endogenous ligands for TRPA1 in butterfly wings are not known. Possible candidates for endogenous ligands include ROS (including hydrogen peroxide), reactive nitrogen species (RNS), reactive carbonyl species, gaseous messengers including nitric oxide and hydrogen sulfide [74,75], polysulfide [76,77], lipids [78], and endogenous cannabinoids [79,80]. These possible ligands should be tested in the future. At this point, use of artificial chemical modulators is probably a reasonable approach to the butterfly wing color pattern formation. On the other hand, there is a possibility that some chemical modulators do not work well due to species differences.

A TRPA1 antagonist, AM0902, tended to enlarge the black core disk area of the ventral forewing eyespots, although another antagonist, AP-18, did not induce any changes. In contrast, a TRPA1 agonist, JT010, decreased the area of the black core disk. These results suggest that TRPA1 is normally responsible for suppressing eyespot enlargement during development. Similar results were obtained in additional trials with different siblings, in which AM0902 increased the outer black ring area of Eyespot E. In the second trial, AP-18 did not induce any significant change, which is also consistent with previous results. Interestingly, in the second trial, JT010 increased the orange ring area of Eyespot E. This result is again consistent with the previous results because an increase in the orange ring area indicates relative shrinkage of the black area of the eyespot. The behavior of the blue focus area was not consistent between the results of AM0902 and JT010; both treatments increased the blue focus area in the second trial. This could be because the focus area is partially independent of the entire eyespot, which is known as the uncoupling rule [40,81]. Furthermore, it should be noted that pharmacological effects of TRPA1 agonists and antagonists are largely dependent on biological systems of interest. For example, it is known that oral administration of AM0902 to rats does not show any effect on hypersensitivity and neuropathic pain models [82]. Both activation and inhibition of calcium levels can be induced by JT010 in CD4^+^ T lymphocytes [83]. Species-specific effects of agonists and antagonists are well known among vertebrate TRPA1 channels [84,85,86,87,88,89,90]. In this context, the behavior of the blue focus area may not be very unreasonable.

As a byproduct of this study, we confirmed the eyespot enlargement effect of DMSO, which has been reported in a previous study [60]. DMSO is known to have numerous biological effects, including immunomodulation, drug delivery and efficacy modulation, and radioprotection [91,92,93], which may be due to its polar and nonpolar groups in a small molecule that can solubilize both hydrophilic and hydrophobic chemicals. We do not know how DMSO enlarges eyespots, but we speculate that when it is injected into pupae, DMSO may change the molecular properties of the inner cuticle to which the epidermal tissue binds, considering that binary adhesion state (attachment/detachment) of epidermal cells to facing pupal cuticle has been proposed as a code for binary color expression (black/white) in scales [54,59,94]. Additionally, DMSO may directly or indirectly quench an inhibitory signal for eyespot formation. Further studies are necessary to resolve this issue.

We obtained partial nucleotide sequences of TRPA1 extracellular and intracellular portions from pupal wing tissues of *J. orithya*, on the basis of which we produced polyclonal anti-TRPA1 antibodies. The antibodies could not induce any changes in the black core disk areas in the ventral forewing eyespots in the first trial, although there may be an increasing tendency in size, if any. The discrepancy between the pharmacological and immunological results is not very surprising, considering that we tested only two portions of TRPA1 as epitopes; our antibodies may not be able to neutralize the function of TRPA1 efficiently, at least in this first trial. Furthermore, it has been reported that monoclonal antibodies against TRPA1 could not completely block the activity of TRPA1 [95,96]. Despite these potential limitations, a surprising phenotype was found in a different sibling group when the anti-TRPA1-Ex antibody was injected: a wing vein defect with disrupted eyespot patterns. The wing vein defect was also associated with irregular midlines and wing surfaces. We believe that the different results between the two sibling groups may be due to the different genetic backgrounds of these sibling groups.

Wing veins play an important role in color pattern formation in butterflies. This is known on the basis of spontaneous wing venation mutants [97]. Without the proper development of wing veins, the border symmetry system (including eyespots) cannot be broken into independent color pattern elements [40,42,98]. That is, wing veins block the lateral expansion of eyespots or break the large band into small chunks in each compartment. When wing vein development is disrupted, the inhibition of eyespot expansion may become less intense. This interpretation is consistent with an increase in eyespot size via the pharmacological inhibition of TRPA1. However, direct action of TRPA1 on eyespot size regulation may also be possible. In addition, we observed the wing surface distortions together with the wing vein defect in the individuals treated with the anti-TRPA1-Ex antibody based on deep contrast imaging. This is probably due to the wing membrane distortions associated with the wing vein defect. Because there are “normal distortions” on the normal wings, the formation of these distortions may be regulated by wing veins. Irregular distortions around eyespots in the wings treated with the anti-TRPA1-Ex antibody suggest that physical distortions of the wing membrane or wing epithelium may be important in normal eyespot development, being consistent with the physical distortion hypothesis [40].

A gene closely related to TRPA1, *painless*, is expressed in the prospective eyespot focus on pupal wing tissues between 3 h and 6 h postpupation in *Bicyclus anynana* [99]. Because there are likely four TRPA members in this butterfly, these TRPA members may be expressed in different regions of pupal wings, for example, one for eyespot focus and another for all epidermal cells. Eyespot focus is known to function as a developmental organizer [42,100,101,102,103,104,105]. Interestingly, many genes involved in wound healing are expressed in eyespots [99]. This is not very surprising, considering that physical damage to pupal wing tissues induces eyespot-like color patterns [106,107,108]. Importantly, the prospective eyespot focus is physically distorted [109,110], which led us to propose the physical distortion hypothesis for color pattern determination [40]. Notably, TRPA1 can be activated via mechanical stimuli [111] and upon wound healing [112], although the mechanosensitivity of TRPA1 may be indirect [111]. The PIEZO channels are probably bona fide mechanoreceptors. That is, for mechanical stimuli, PIEZO is probably the direct receptor [113,114], and TRPA1 is then activated indirectly [111]. Recently, we showed that PIEZO1 may be responsible for eyespot formation in butterfly wings [60]. PIEZO1 appears to suppress black area formation [60]. TRPA1 also appears to suppress black area formation, according to the present study, suggesting that PIEZO1 and TRPA1 may cooperate in butterfly wing color pattern formation.

High and low temperatures are also important candidates for TRPA1-activating stimuli in butterfly wings because wings exhibit phenotypic plasticity in response to seasonal temperatures and sudden temperature changes (i.e., cold shock and heat shock). Although they are known to be mediated via hormones, wing epidermal cells may also sense temperature directly. As stress-inducing chemicals such as thapsigargin, plant secondary metabolites may be toxic to insects and may cause color pattern changes. However, herbivorous insects are often well adapted to toxic chemicals from the host plant. As DMSO increases the eyespot size, hydrogen peroxide and other reactive oxygen species may activate TRP channels [24,115]. Further studies are necessary to understand the functions of TRP and PIEZO receptors and their relationships with other genes, such as Wnt family members [116,117,118,119,120,121,122], in butterfly wing color pattern development.

In this study, we showed that TRPA1 is expressed in wing tissues by RT-PCR and subsequent DNA sequencing, based on which we assume that TRPA1 is distributed throughout the wing surface to respond to endogenous and exogenous signals at the level of single cell, but its experimental evidence is lacking. Localization of TRPA1 on the wing tissue may be performed via immunohistochemistry, but the immunohistochemical detection of cell-surface membrane proteins including TRPA1 may not be technically demanding. Indeed, we performed a novel high-resolution localization method called CLAMP (catalyzed labeling for signal amplification) [123] for TRPA1, but it has not been successful due to high background noise. An alternative may be proteomic analyses [124,125]. Additionally, in accordance with the previous studies [72,73], physiological activities of epidermal cells may be investigated via calcium imaging in response to agonists and antagonists in the future.

## 4. Materials and Methods

### 4.1. Butterfly Rearing

Throughout this study, we focused on females of the blue pansy butterfly *J. orithya* (Linnaeus, 1758) because this species is sexually dimorphic. We collected wild female butterflies of *J. orithya* from the Nishihara Campus of the University of the Ryukyus and some parks in Okinawa-jima Island, Japan. We placed these butterflies in a cubic glass tank (300 mm × 300 mm × 300 mm). We also placed the host plant leaves of *Phyla nodiflora* in the same tank for egg laying. More than one individual was confined to a tank, but we often experienced that only one or a few females produced numerous eggs. A group of eggs produced in this way was considered a sibling group. After hatching, the larvae were reared with leaves of *Plantago asiatica*, another natural host plant. The entire process was conducted at ambient temperature (approximately 27 °C) under L16:D8 light conditions.

### 4.2. Chemical Treatments

We performed chemical injections on one side of the abdomen of each pupa via an Ito microsyringe MS-05 (Fuji, Shizuoka, Japan). This treatment was performed within 5 h after pupation. The following three chemical modulators of TRPA1 were used: AM0902 (1.49 mg/mL; Cayman Chemical, Ann Arbor, MI, USA, and 1.67 mg/mL; Toronto Research Chemicals, Toronto, ON, Canada), AP-18 (20.97 mg/mL; Cayman Chemical), and JT010 (0.71 or 7.10 mg/mL; Selleckchem, Houston, TX, USA). They were dissolved in DMSO (FUJIFILM Wako Chemicals, Osaka, Japan). We injected 2.0 μL per pupa, with the exception of the anti-TRPA1 extracellular (anti-TRPA1-Ex) antibody and its control anti-spike P1 antibody (4 μL) in the first trial (Table 1). The anti-TRPA1 intracellular (anti-TRPA1-In) antibody was mixed with a protein delivery reagent ProteoCarry (Funakoshi, Tokyo, Japan) for intracellular delivery before injection in accordance with a previous study [126]. We recorded the total number of treated individuals, the number of individuals with successful eclosion (males and females), and the percentages of successful eclosion among the treated individuals (eclosion rate or ER) (Table 1). The eclosion rate was used to understand the toxicity level of a chemical very roughly.

Statistical comparisons between the experimental and control groups were made within the same sibling group, considering color pattern variations among different sibling groups in this species. For records, images of all individuals used in this study are shown in Supplementary Figures S1–S5.

### 4.3. Image Analyses

**Figure 11:**
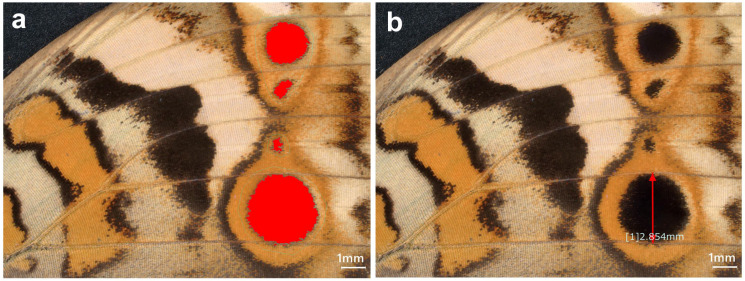
Image quantification of eyespots. (**a**) The area values of the eyespot core disks on the ventral forewing, as indicated in red. (**b**) Wing compartment width value as indicated by the double-headed arrows.

We compared the area values of the given eyespot structures between the experimental and control groups. We measured eyespot area values via image analyses. An example is shown in Figure 11. In this example, the black core disk areas of the ventral forewing eyespots were measured together as a single value (collective measurements). Alternatively, eyespots were measured individually (individual measurements): anterior and posterior dorsal forewing eyespots (Eyespots A and B in Figure 1), anterior and posterior ventral forewing eyespots (Eyespots C and D in Figure 1), and anterior and posterior dorsal hindwing eyespots (Eyespots E and F in Figure 1).

A Keyence Digital Microscope VHX-7000 (Osaka, Japan) was used for these image-based area measurements. The built-in software automatically recognized the area to be measured (region of interest, ROI) after setting a threshold value, but the automatic ROIs were visually confirmed to be correct. To obtain a relative size that was not affected by individual wing size, the area value was divided by the squared value of the width of the compartment in which the eyespot was present for individual measurements. For collective measurements, the width of the Eyespot D compartment was used. To do so, we measured a line on the central structural scales perpendicular to the wing veins. If necessary, the area values were corrected by the correction factors for the DMSO effect [60]. For convenience, we set the mean value of the control group as one. With these area values, bisided unpaired *t* tests were performed after the *F* test for equal variance using JSTAT version 16.1 (Yokohama, Japan) and Microsoft Excel (Microsoft Office 365).

Deep contrast images were also obtained using the opt-SEM (optical shadow effect mode) function of a Keyence Digital Microscope VHX-7000. This function detects and expresses height information (at the resolution of a few micrometers) in final images. This function is based on multi-directional illumination, which produces multiple images with different light intensities, shades, and reflections. These multiple images were subjected to reflectance/shift analysis and were integrated into a final image in which height differences were easily recognizable to human eyes.

### 4.4. RT-PCR and DNA Sequence Analyses

The expression of TRPA1 at the mRNA level was examined via RT-PCR using a Veriti200 Veriti96-Well thermal cycler (Applied Biosystems, Foster City, CA, USA). To do so, fresh pupae were frozen within 10 h postpupation at −80 °C, and then their pupal wing tissues were dissected with forceps and scissors. We used NucleoSpin RNA Plus (Takara Bio, Kusatsu, Shiga, Japan) to extract RNA from the dissected tissues in accordance with the manufacturer’s protocol. RT-PCR was performed using a PrimeScript II High-Fidelity RT-PCR Kit (Takara Bio). We used the following RT reaction conditions: 30 °C (10 min), 42 °C (30 min), 95 °C (5 min), and 4 °C. For PCR, we used TaKaRa Ex Premier DNA Polymerase (Takara Bio). For the intracellular target, we used the following conditions for both the first and second (nested) PCR cycles: 94 °C (1 min) and then 35 cycles of 98 °C (10 s), 55 °C (15 s), and 68 °C (30 s). For the extracellular target, we used the following conditions: 94 °C (1 min) and then 35 cycles of 98 °C (10 s), 55 °C (15 s), and 68 °C (30 s) for the first PCR cycles, and 94 °C (1 min) and then 35 cycles of 98 °C (10 s), 60 °C (15 s), and 68 °C (30 s) for the second (nested) PCR cycles.

We designed primers based on TRPA1 mRNA sequences from the painted lady butterfly *Vanessa cardui* (NCBI Reference Sequence ID: XM_047121213.1, Gene ID: 124543143). This is because the genus *Vanessa* is reasonably close to the genus *Junonia* in Nymphalidae [127,128,129]. The PCR primers were designed to cover different exons to prevent the amplification of genomic DNA. The following PCR primers were obtained from Eurofins Genomics (Tokyo, Japan). The TRPA1 extracellular primers for the first PCR were 5′-TATCTATGGGATGTCGCTTGAGTTAC-3′ and 5′-GAACATCACTACGTATATTCCGA CTTG-3′ (expected product size: 926 bp from *V. cardui*). The TRPA1 extracellular primers used for the second (nested) PCR were 5′-GAGTTACAATAACCTGGACTTGAGCGC-3′ and 5′-CTACGTATATTCCGACTTGATCGAAACG-3′ (expected product size: 899 bp from *V. cardui*). The TRPA1 intracellular primers used for the first PCR were 5′-GTGTGCGCAGGGGGCTCTCGAGATTATCGA-3′ and 5′-TCCATCTTTATCCGACTG ATCAAGTAAGTG-3′ (expected product size: 841 bp from *V. cardui*). The TRPA1 intracellular primers used for the second (nested) PCR were 5′-GGGCTCTCGAGATTATCGAGCTAATGTTTC-3′ and 5′-CAAGTAAGTGTGAATGGACCGAGTGTAG-3′ (expected product size: 786 bp from *V. cardui*).

For negative controls of RT-PCR, an RNA sample was treated without the addition of reverse transcriptase (but with the addition of ultrapure water instead), but the other steps were identical to those of the experimental samples. For the positive control of RT-PCR, an RNA sample and corresponding primers provided in the PrimeScript II High Fidelity RT-PCR Kit were used according to the manufacturer’s protocol. The expected size of the positive control RT-PCR product was 462 bp.

The PCR products, together with a DNA size marker, FastGene 100 bp DNA Ladder Plus (NIPPON Genetics, Tokyo, Japan), were subjected to agarose gel electrophoresis (1.5% agarose in TAE, 100 V). The DNA was stained with RedSafe Nucleic Acid Staining Solution (iNtRON Biotechnology, Seongnam-si, Gyeonggi-do, Republic of Korea) and was observed and photographed via a GelDoc XR Plus Imaging System (Bio-Rad Laboratories, Hercules, CA, USA). The original gel image is presented in Supplementary Figure S6. The positive PCR products were purified with NucleoSpin Gel and PCR Clean-up (Takara Bio) and were sequenced in both directions via direct Sanger dideoxy sequencing at Eurofins Genomics. The sequences were examined with BLAST (Basic Local Alignment Search Tool; https://blast.ncbi.nlm.nih.gov/Blast.cgi; accessed on 1 July 2025) and confirmed to be a novel TRPA1 gene from *J. orithya*. The cDNA (mRNA) sequences obtained in the present study were then deposited in GenBank (GenBank Accession Numbers: PX675202 for the extracellular site and PX675203 for the intracellular site).

### 4.5. Antibodies

On the basis of the conceptually translated sequences of *J. orithya* TRPA1, we designed two peptide epitopes, one from the intracellular site (CHRDHSGPQPAPSSGD) and the other from the extracellular site (NSTDNSIKKDFETMC). A cysteine residue was added to the amino or carboxyl terminus of the synthetic peptides for KLH (keyhole limpet hemocyanin) conjugation via an MBS (*m*-malrimidobenzoyl-*N*-hydroxysuccinimide ester) linker. The Ellman method was used to confirm successful conjugation. Peptide synthesis and subsequent polyclonal antibody production were performed at the Sapporo Laboratory of Cosmo Bio (Tokyo, Japan) as the Fast Antibody Plus service package. Peptides were produced via the Fmoc solid-phase synthesis method. Peptides were checked for their purity and integrity via high-performance liquid chromatography (HPLC) and time-of-flight mass spectrometry (TOF-MS) (Shimadzu AXIMA Confidence 2.9.3, Kyoto, Japan) at 220 nm.

Immunization with a KLH-conjugated peptide was performed at days 0, 14, 28, and 42 in a Japanese White rabbit. For the first injection on day 0, 400 μg was injected. For the subsequent injections, the quantity of injections was 200 μg. Blood samples (2 mL) were obtained on days 0 and 35. All blood (40–45 mL) was taken and subjected to antibody purification on day 56. Peptide affinity column chromatography was performed to purify specific antibodies from blood serum (10 mL). ProClin 300 (Sigma-Aldrich, Merck, Rahway, NJ, USA) was added to the purified antibody as a preservative. To assess the antibody concentration, ELISA was performed using an anti-rabbit IgG conjugated with alkaline phosphatase as a secondary antibody. The final concentrations of the TRPA1 intracellular and extracellular antibodies were estimated to be 0.98 mg/mL and 0.68 mg/mL, respectively. The control anti-spike P1 antibody was made in the same way as the Fast Antibody service package in the Sapporo Laboratory of Cosmo Bio and was used for the study of SARS-CoV-2 infection and vaccination from the viewpoint of molecular mimicry [130]. The peptide epitope of the control antibody was taken from the spike protein of SARS-CoV-2 [130]. The final concentration of the anti-spike P1 antibody was 0.28 mg/mL [130].

## 5. Conclusions

Here, we showed the possible involvement of TRPA1 in butterfly wing eyespot color pattern formation in *J. orithya* via pharmacological assays. TRPA1 is likely important for eyespot formation and wing vein development. If mechanical signals are involved in color pattern determination, mechanical signal reception may be executed by TRPA1 together with PIEZO1, but TRPA1 may integrate multimodal signals from the environment, including temperature, light, and chemical signals, together with mechanical signals. Mechanical signals may be produced de novo during development. Alternatively, mechanical signals may be produced via physical damage. Either way, TRPA1 may be able to function to produce long-range intracellular calcium waves that run laterally and relatively slowly on wing tissue. The developmental functions of TRPA1 in nonneuronal cells and tissues will be explored further not only in butterfly wings but also in other animal systems.

## Figures and Tables

**Table 1:** Number of individuals used for the injections in this study.

Trial (Sibling) Number	Treatment (Concentration, Injection Volume)	Total Number of Treated Individuals (Both Sexes)	Number of Individuals with Successful Eclosion (Both Sexes)	Percentage of Successful Eclosion (Eclosion Rate, ER)	Number of Females with Successful Eclosion
#1	No treatment	40	31	78%	24
	DMSO (2 μL)	34	25	74%	18
	AM0902 (1.49 mg/mL, 2 μL)	30	26	87%	11
	AP-18 (20.97 mg/mL, 2 μL)	28	11	39%	9
	Anti-TRPA1-Ex antibody (no dilution, 4 μL)	36	33	92%	15
	Anti-spike P1 antibody (no dilution, 4 μL)	28	26	93%	19
	Anti-TRPA1-In antibody + ProteoCarry (1:8, 2 μL)	6	5	83%	4
	Anti-spike P1 antibody + ProteoCarry (1:8, 2 μL)	7	6	86%	4
#2	No treatment	39	30	77%	13
	DMSO (2 μL)	41	27	66%	11
	JT010 (7.1 mg/mL, 2 μL)	41	29	71%	13
	Anti-TRPA1-In antibody + ProteoCarry (1:8, 2 μL)	26	23	89%	9
	Anti-spike P1 antibody + ProteoCarry (1:8, 2 μL)	18	15	83%	8
#3	No treatment	58	54	93%	21
	AM0902 (1.67 mg/mL, 2 μL)	41	13	32%	4
	JT010 (0.71 mg/mL, 2 μL)	30	8	27%	6
#4	No treatment	41	41	100%	22
	AP-18 (20.97 mg/mL, 2 μL)	25	20	80%	15
#5	No treatment	18	18	100%	11
	Anti-TRPA1-Ex antibody (no dilution, 2 μL)	34	34	100%	20

## Data Availability

The original contributions presented in this study are included in the article and Supplementary Materials. Further inquiries can be directed to the corresponding author.

## Supplementary Materials

The following supporting information can be downloaded at: https://www.mdpi.com/article/10.3390/ijms27031420/s1.

## Abbreviations

The following abbreviations are used in this manuscript:

TRPA1	Transient receptor potential ankyrin 1
Ex	Extracellular site
In	Intracellular site
*J.*	*Junonia*
FB28	Fluorescent brightener 28
DMSO	Dimethyl sulfoxide
RT-PCR	Reverse transcriptase-polymerase chain reaction
ROS	Reactive oxygen species
BLAST	Basic local alignment search tool
