# Supplementary material for: TRPA1 for Butterfly Eyespot Formation
Source: Int J Mol Sci. 2026 Jan 30;27(3):1420. doi: 10.3390/ijms27031420 (PMC12898838; doi:10.3390/ijms27031420)

TRPA1 for Butterfly Eyespot Regulation

Momo Ozaki [1] and Joji M. Otaki [1,*]

[1] The BCPH Unit of Molecular Physiology, Department of Chemistry, Biology and Marine Science, Faculty of Science, University of the Ryukyus, Nishihara, Okinawa 903-0213, Japan.
* Correspondence: otaki@cs.u-ryukyu.ac.jp, Tel. : +81-98-895-8557

Supplementary Figure S6. Original gel image for Figure 7.

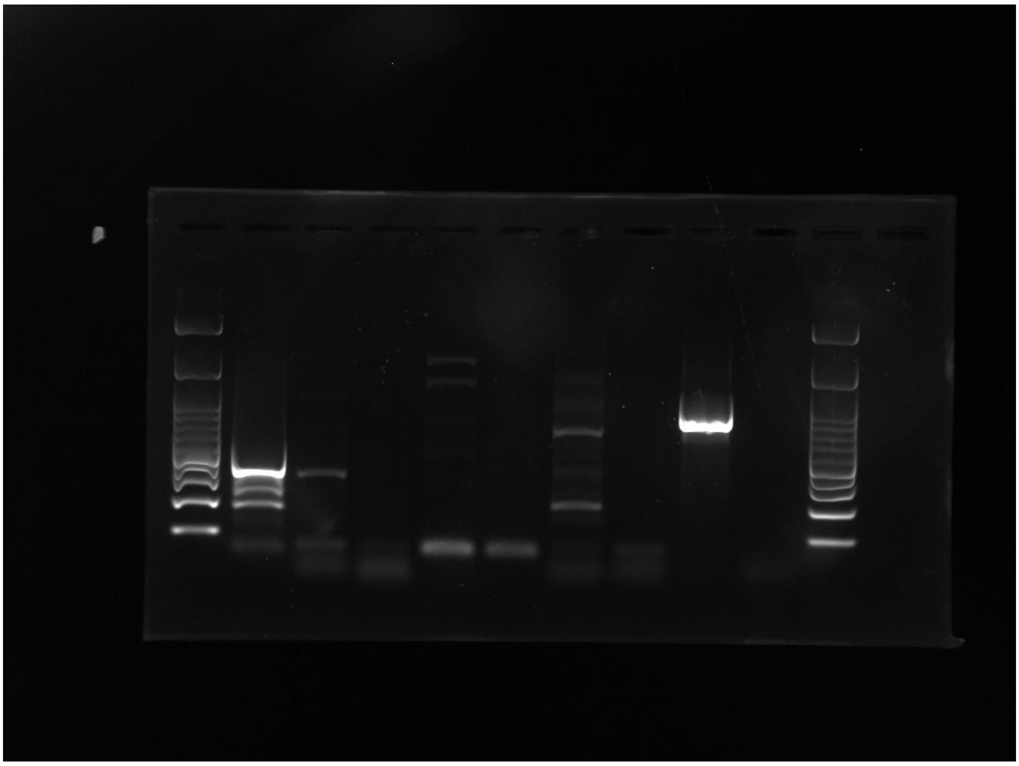

Supplement: Supplementary file 1 [file ijms-27-01420-s001.zip › TRPA1 Supplementary Figure S6.pdf]
