# Supplementary material for: TRPA1 for Butterfly Eyespot Formation
Source: Int J Mol Sci. 2026 Jan 30;27(3):1420. doi: 10.3390/ijms27031420 (PMC12898838; doi:10.3390/ijms27031420)

TRPA1 for Butterfly Eyespot Formation

Momo Ozaki [1] and Joji M. Otaki [1,*]

[1] The BCPH Unit of Molecular Physiology, Department of Chemistry, Biology and Marine Science, Faculty of Science, University of the Ryukyus, Nishihara, Okinawa 903-0213, Japan.
[*] Correspondence: otaki@cs.u-ryukyu.ac.jp, Tel. : +81-98-895-8557

Supplementary Figure S4. Wings of the trial (sibling) No. 4. Shown are all females. The dorsal side (left) and the ventral side (right) are shown. (**a**) No treatment (*n* = 22). (**b**) AP-18 (20.97 mg/mL) treatment (*n* = 15).

(**a**) No treatment (*n* = 22).

No. 1

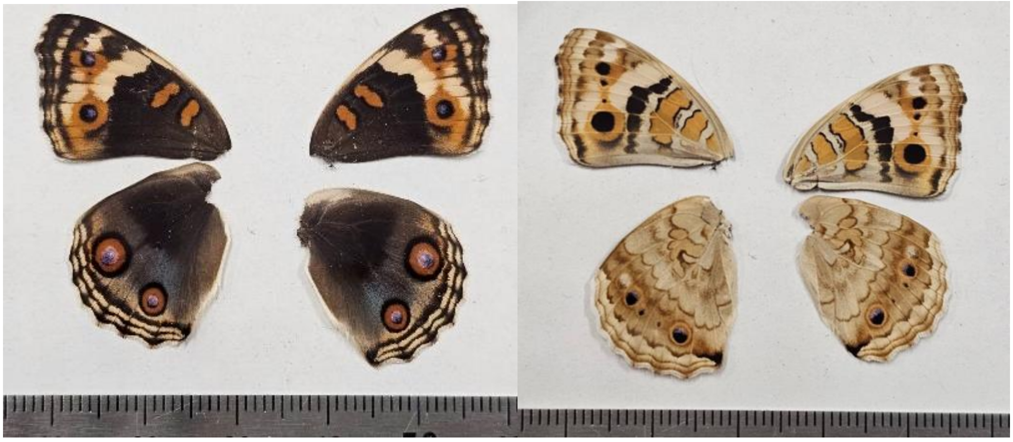

No. 2

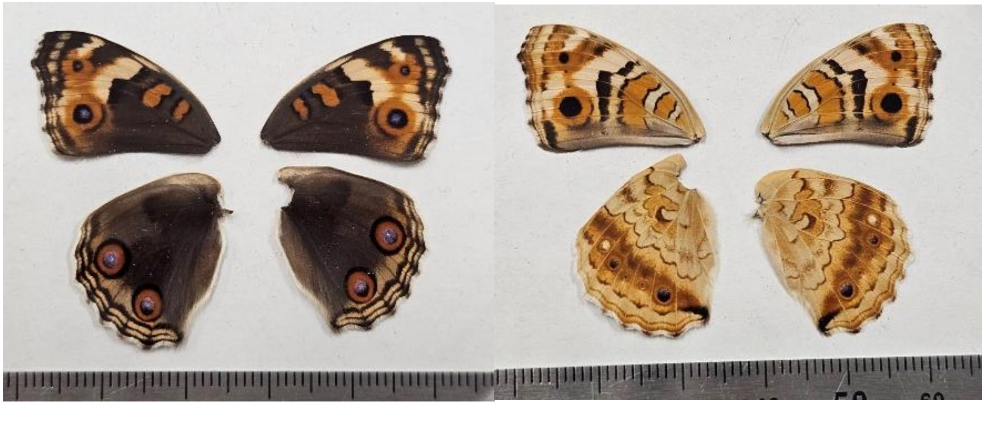

No. 3

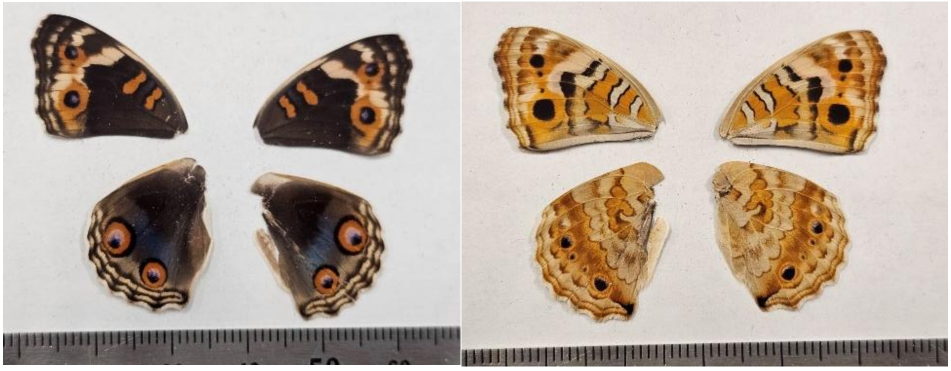

No. 4

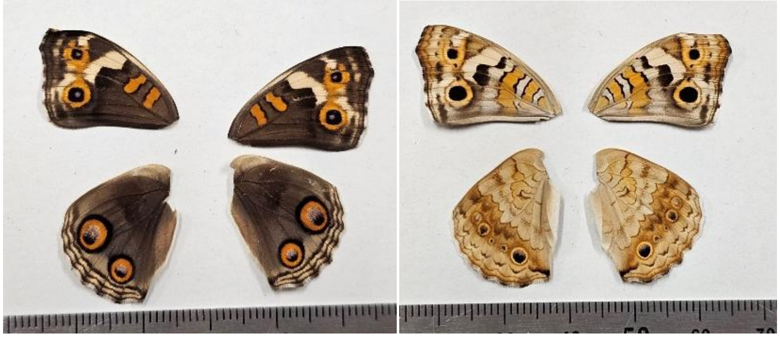

No. 5

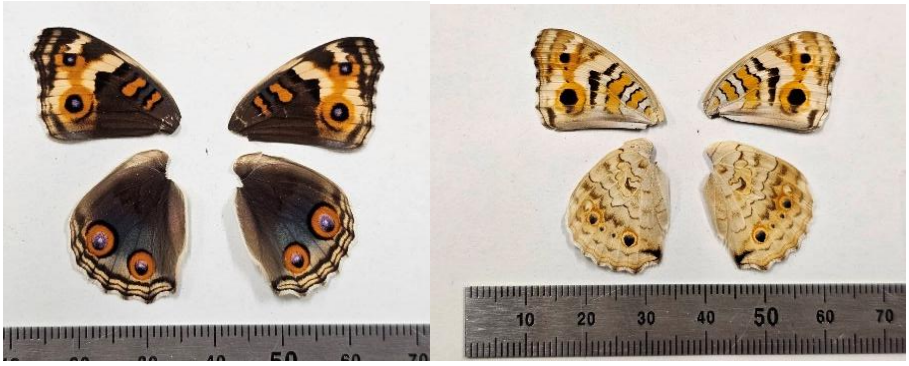

No. 6

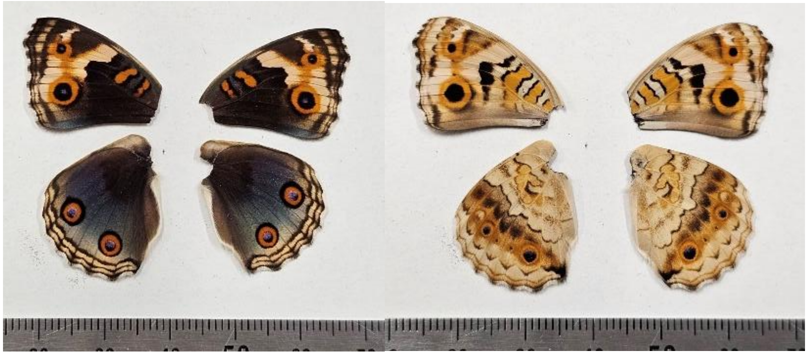

No. 7

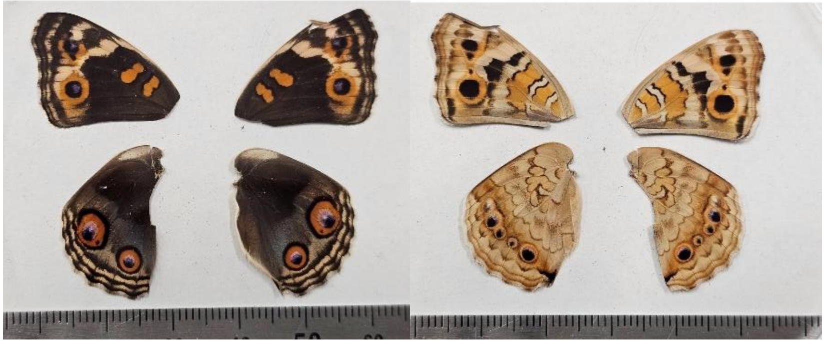

No. 8

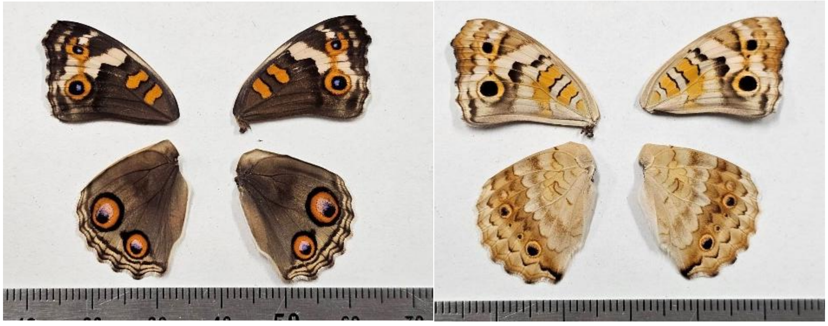

No. 9

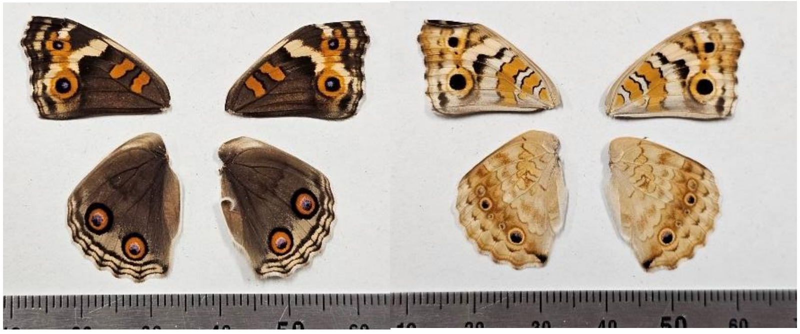

No. 10

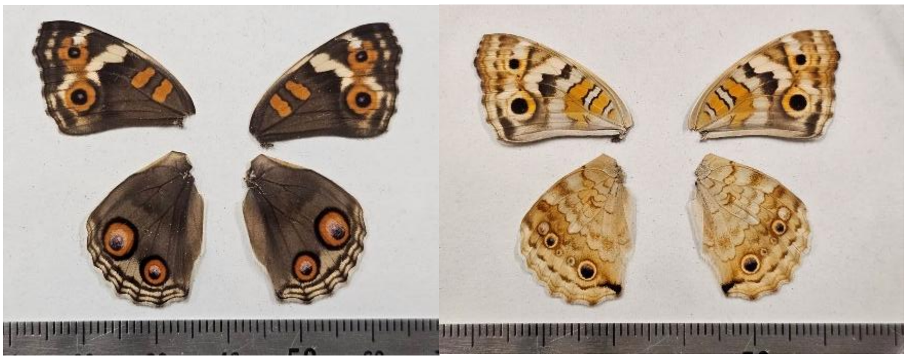

No. 11

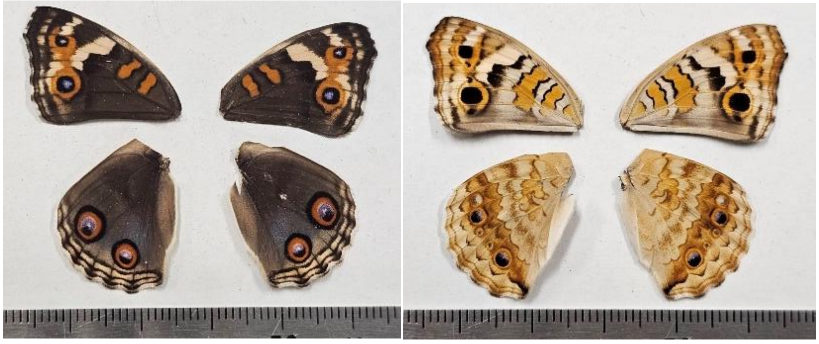

No. 12

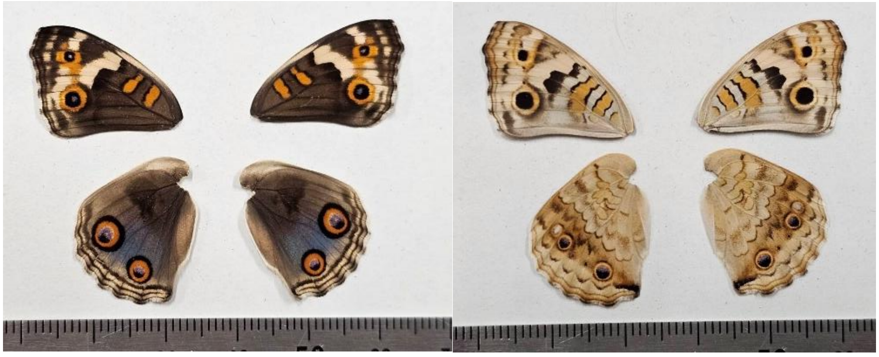

No. 13

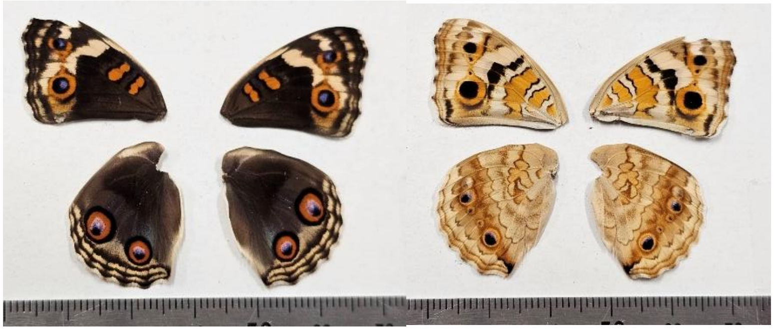

No. 14

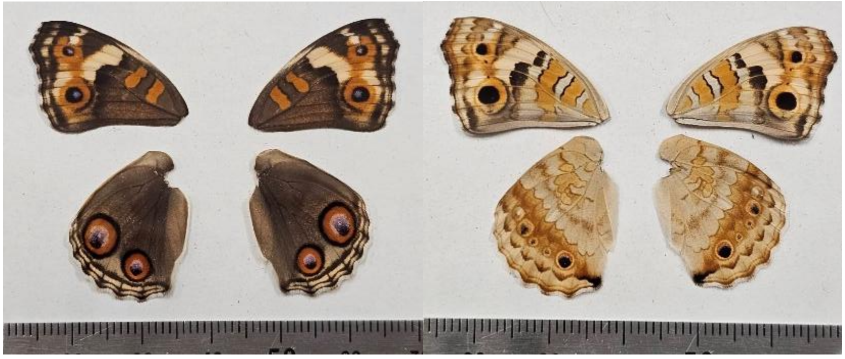

No. 15

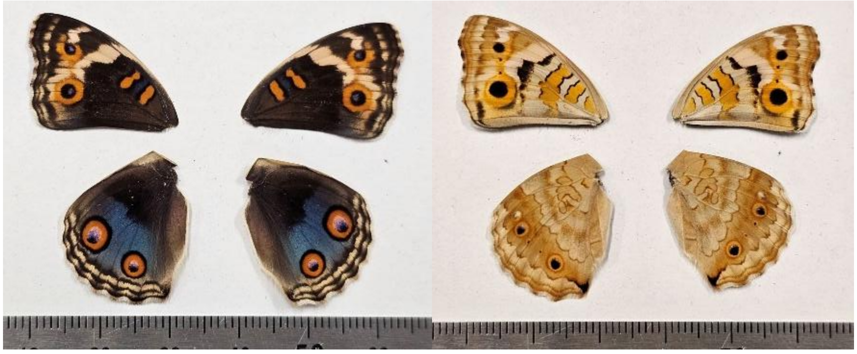

No. 16

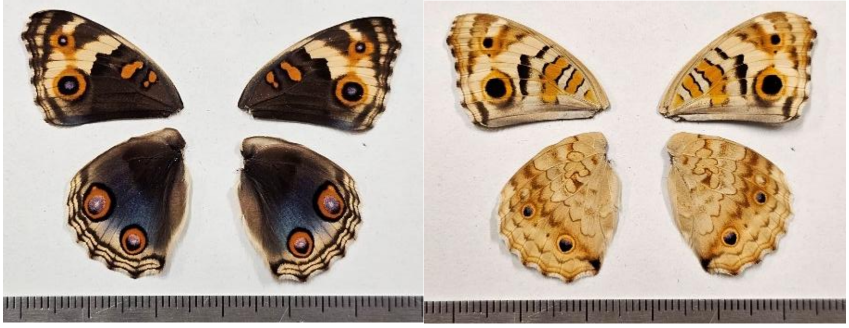

No. 17

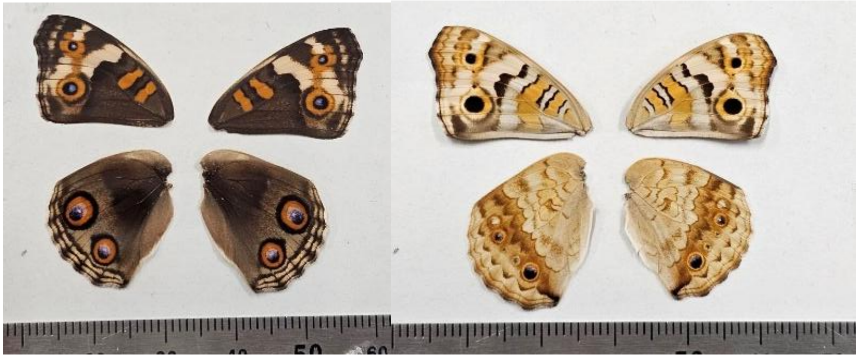

No. 18

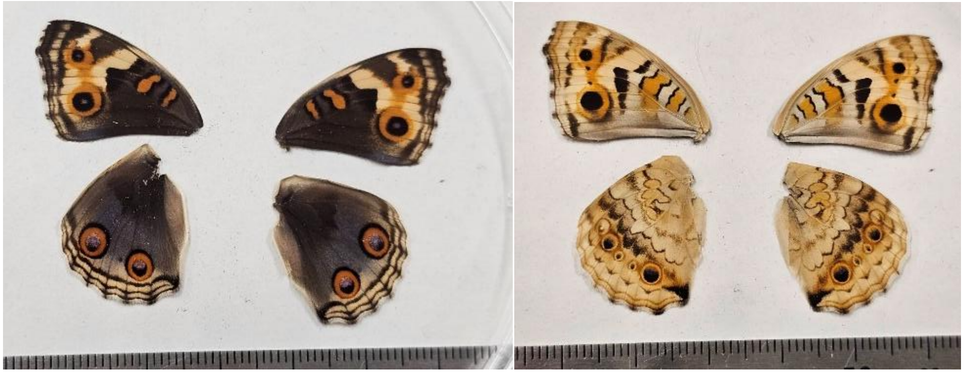

No. 19

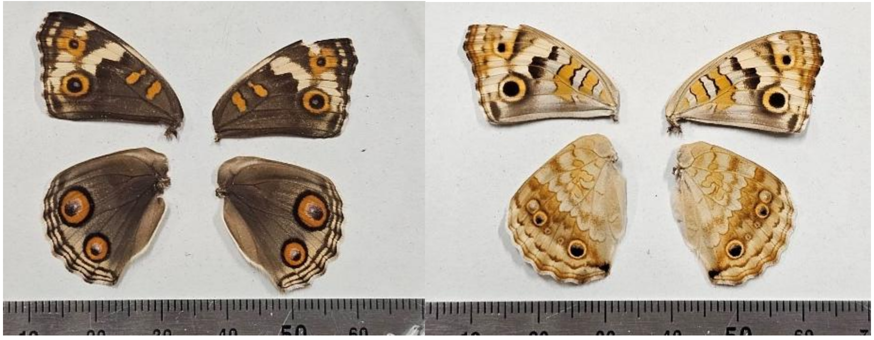

No. 20

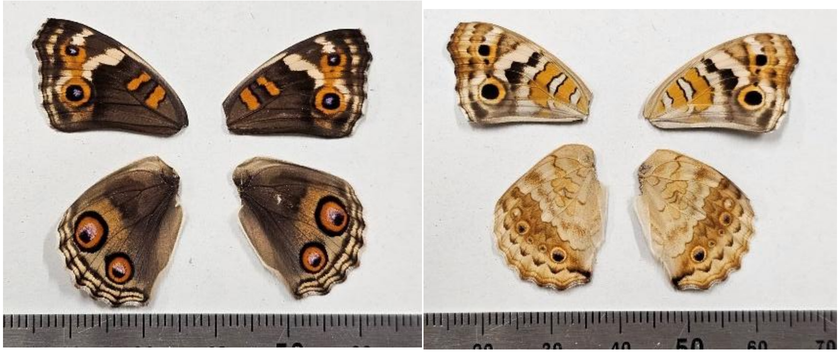

No. 21

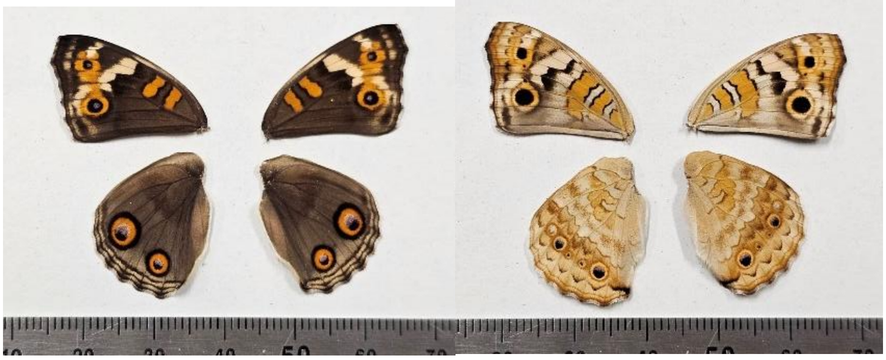

No. 22

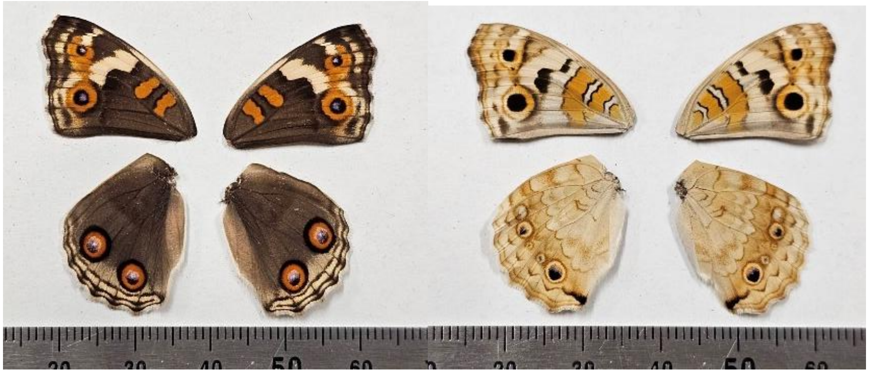

(**b**) AP-18 (20.97 mg/mL) treatment (*n* = 15).

No. 1

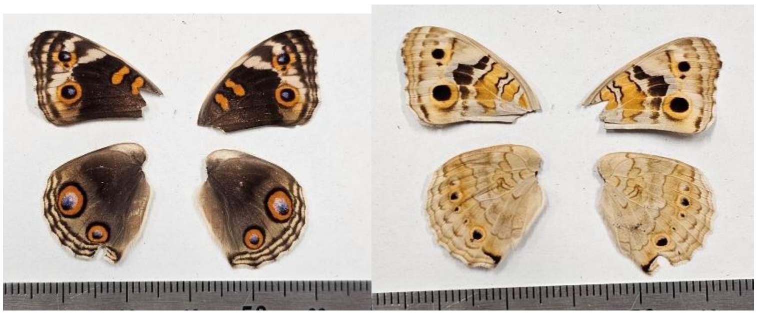

No. 2

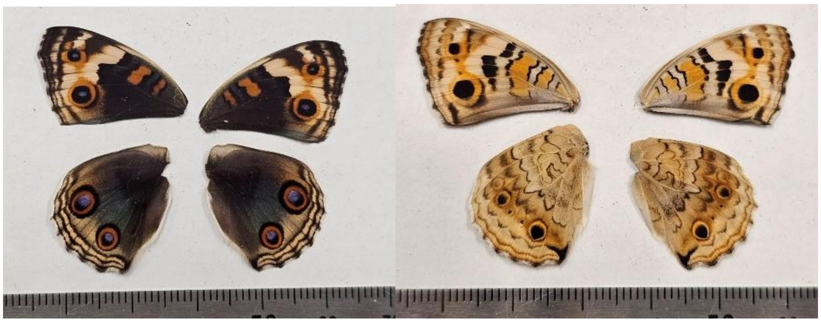

No. 3

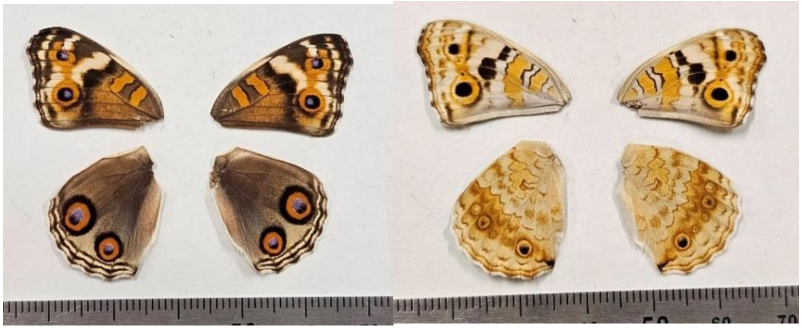

No. 4

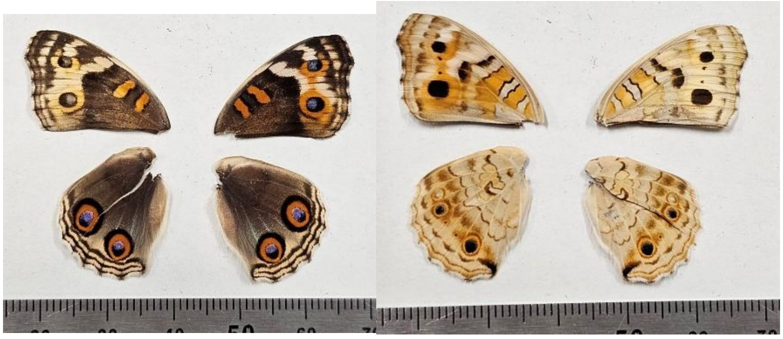

No. 5

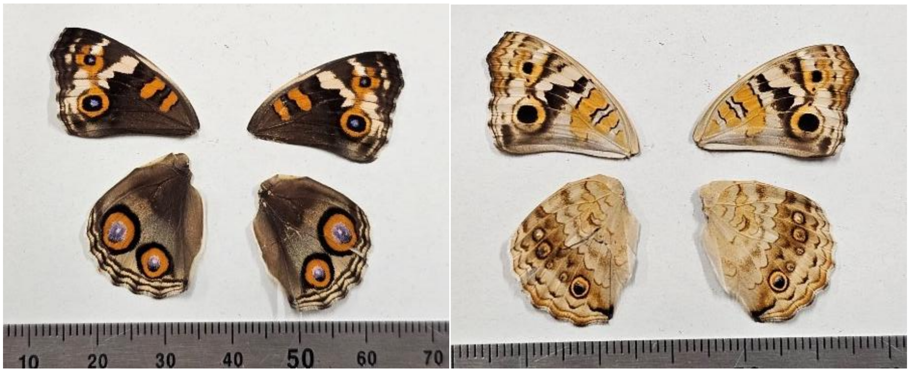

No. 6

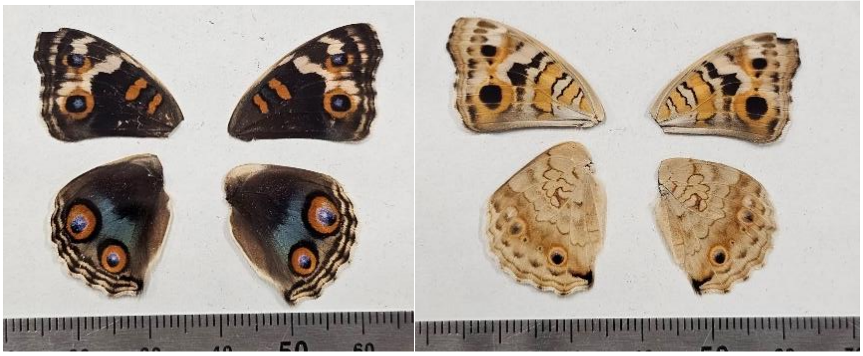

No. 7

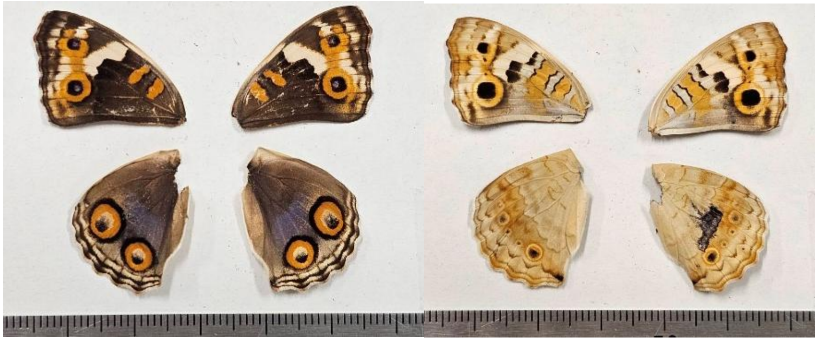

No. 8

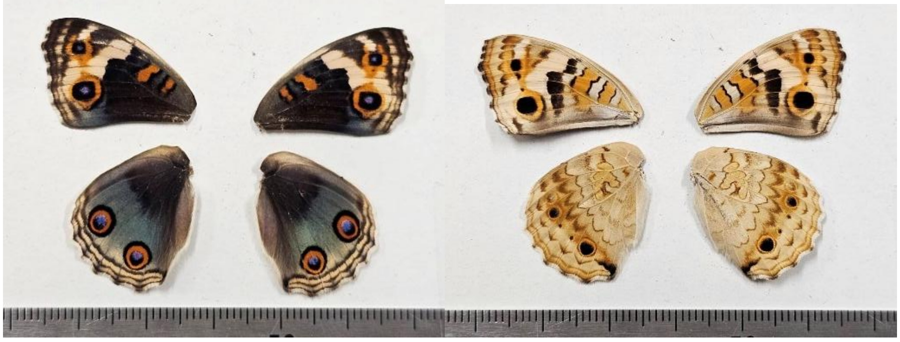

No. 9

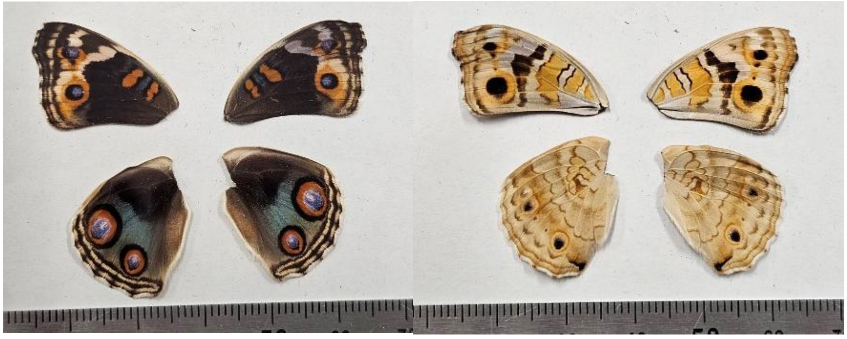

No. 10

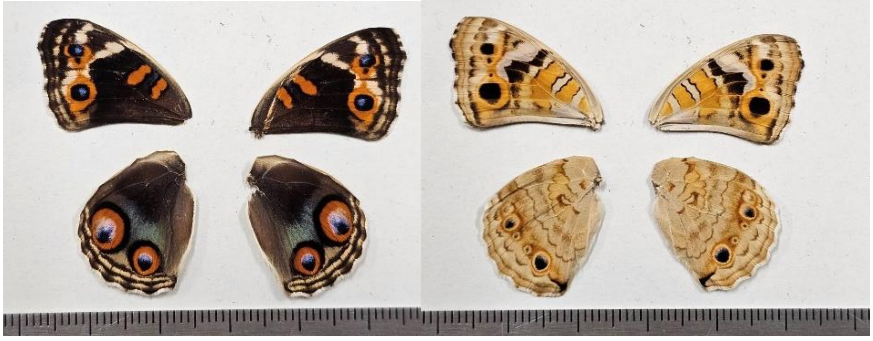

No. 11

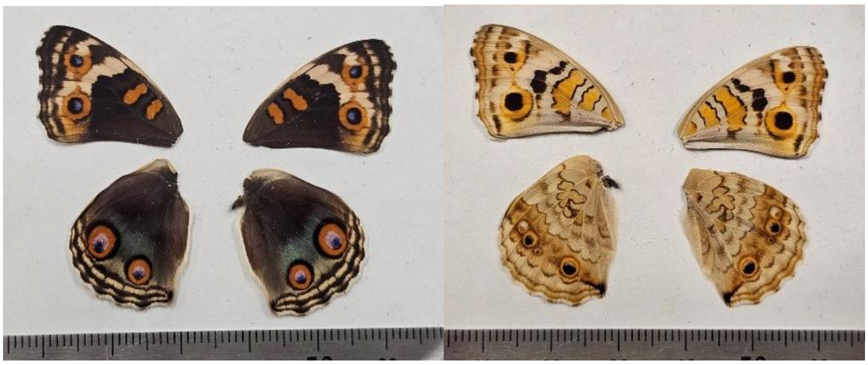

No. 12

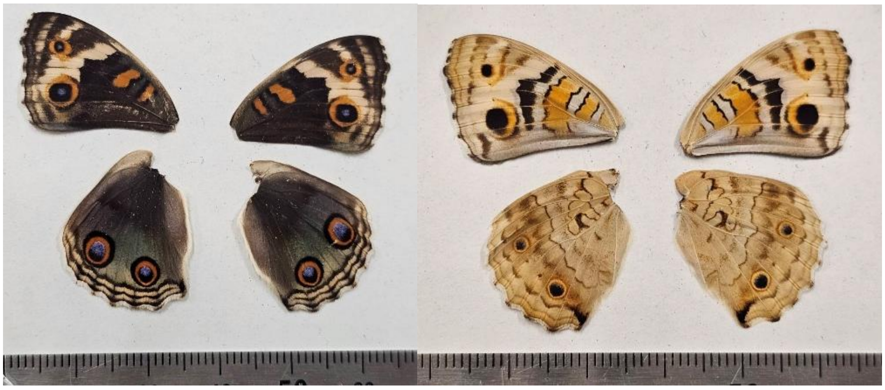

No. 13

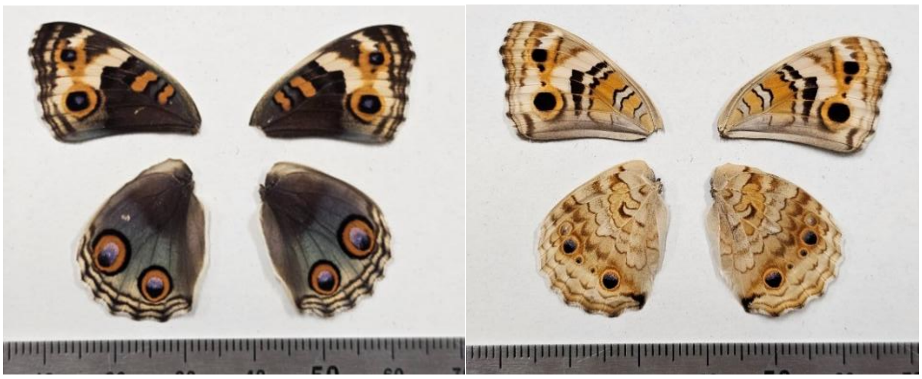

No. 14

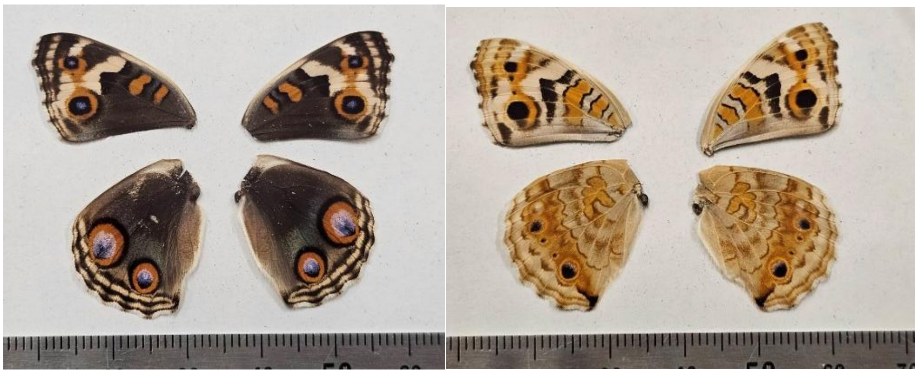

No. 15

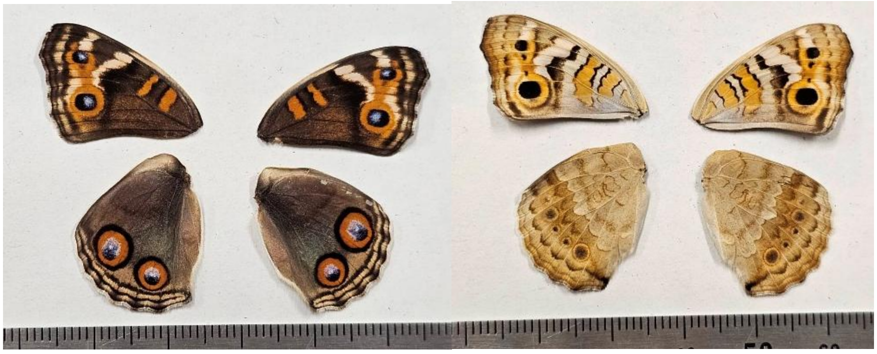

Supplement: Supplementary file 1 [file ijms-27-01420-s001.zip › TRPA1 Supplementary Figure S4.pdf]
