# Supplementary material for: TRPA1 for Butterfly Eyespot Formation
Source: Int J Mol Sci. 2026 Jan 30;27(3):1420. doi: 10.3390/ijms27031420 (PMC12898838; doi:10.3390/ijms27031420)

TRPA1 for Butterfly Eyespot Formation

Momo Ozaki [1] and Joji M. Otaki [1,*]

[1] The BCPH Unit of Molecular Physiology, Department of Chemistry, Biology and Marine Science, Faculty of Science, University of the Ryukyus, Nishihara, Okinawa 903-0213, Japan.
[*] Correspondence: otaki@cs.u-ryukyu.ac.jp, Tel. : +81-98-895-8557

Supplementary Figure S1. Wings of the trial (sibling) No. 1. Shown are all females. The dorsal side (left) and the ventral side (right) are shown. (**a**) No treatment (*n* = 24). (**b**) DMSO treatment (*n* = 18). (**c**) AM0902 treatment (*n* = 11). (**d**) AP-18 treatment (*n* = 9). (**e**) Anti-TRPA1-Ex antibody treatment (*n* = 15). (**f**) Anti-spike P1 antibody (*n* = 19). (**g**) Anti-TRPA1-In antibody plus ProteoCarry treatment (*n* = 4). (**h**) Anti-spike P1 antibody plus ProteoCarry treatment (*n* = 4).

(**a**) No treatment (*n* = 24).

No. 1

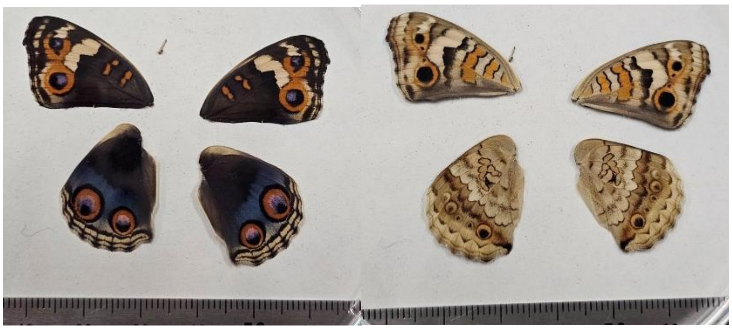

No. 2

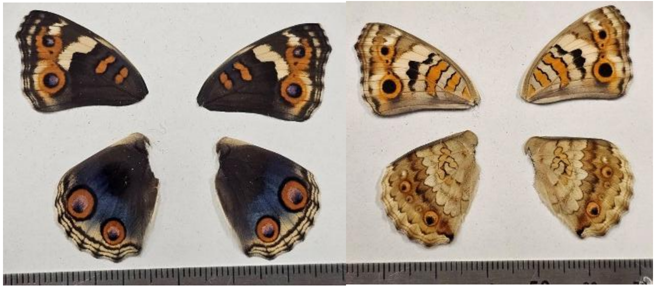

No. 3

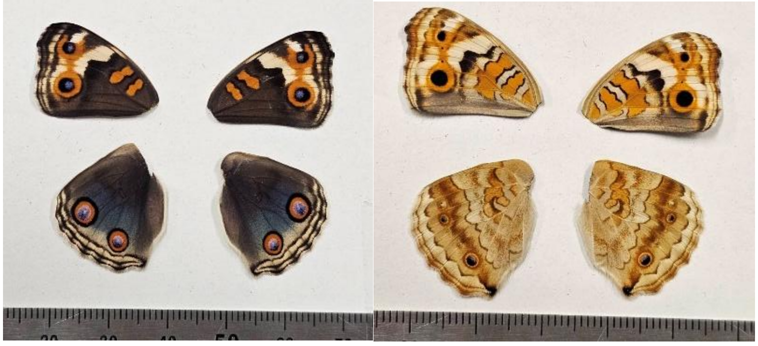

No. 4

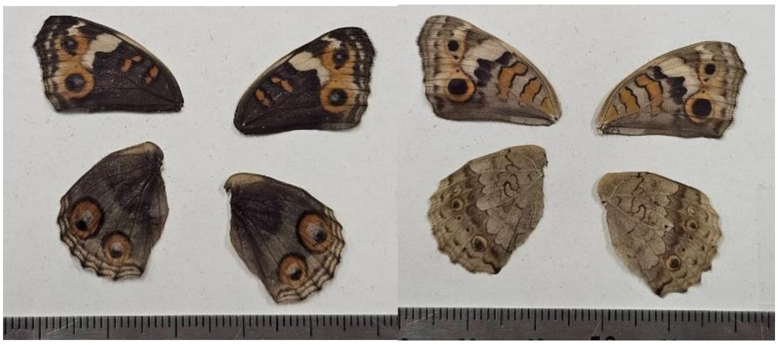

No. 5

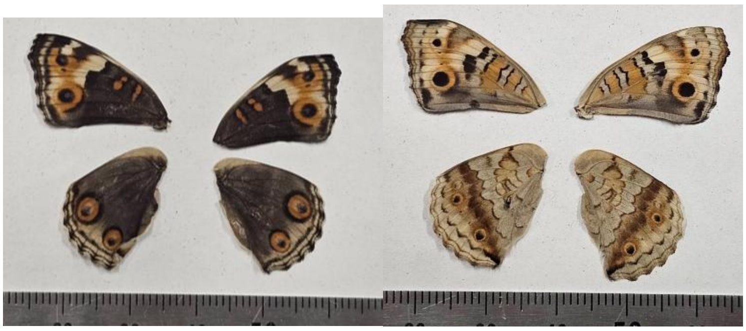

No. 6

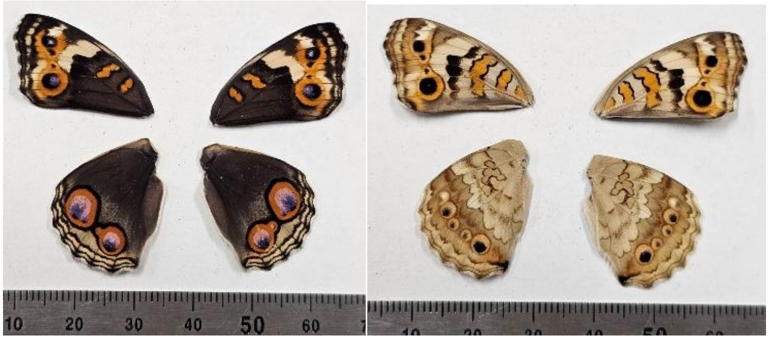

No. 7

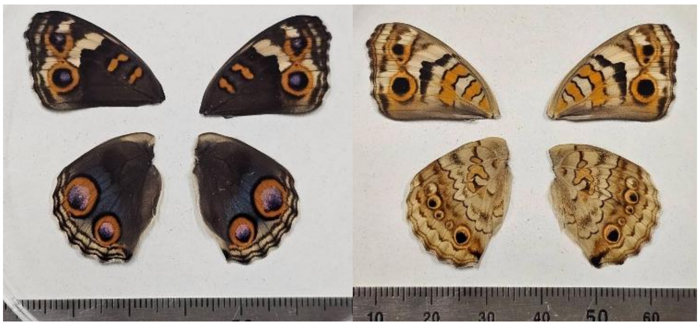

No. 8

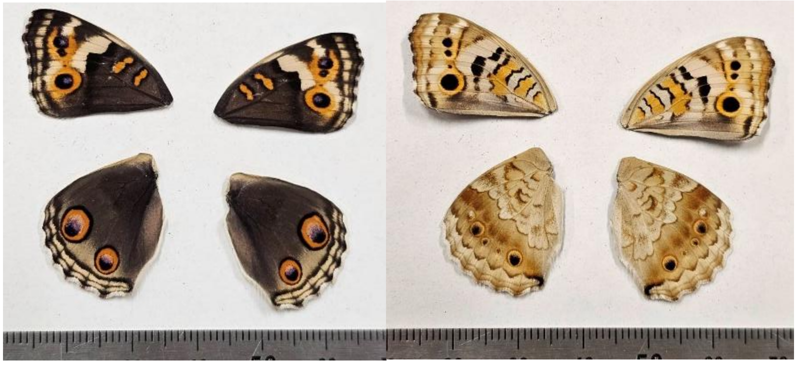

No. 9

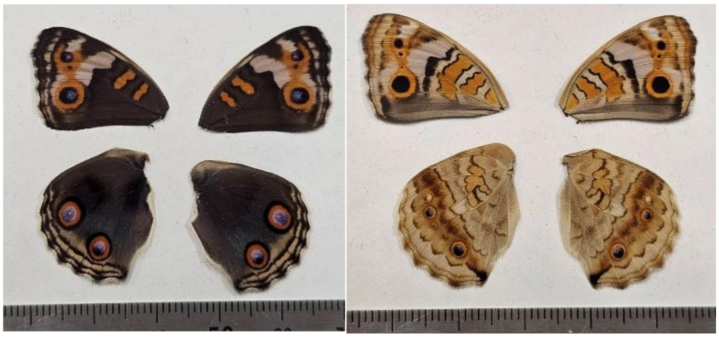

No. 10

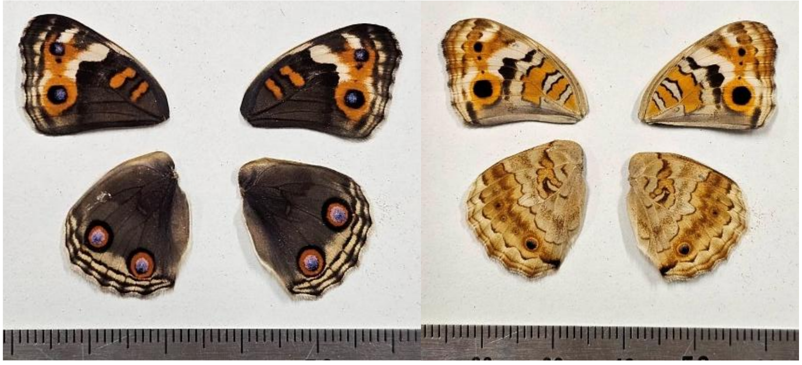

No. 11

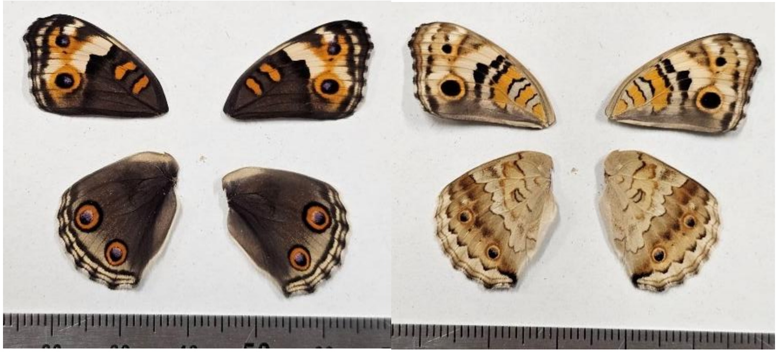

No. 12

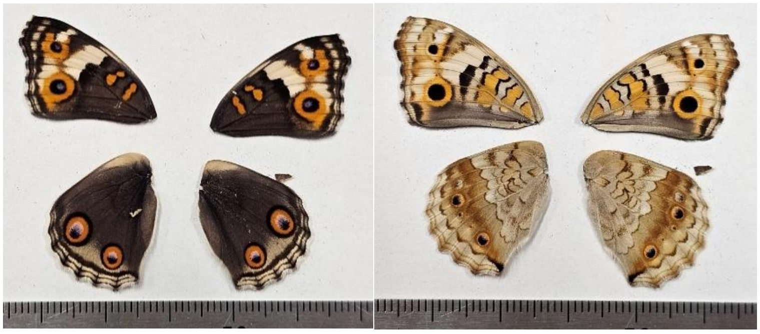

No. 13

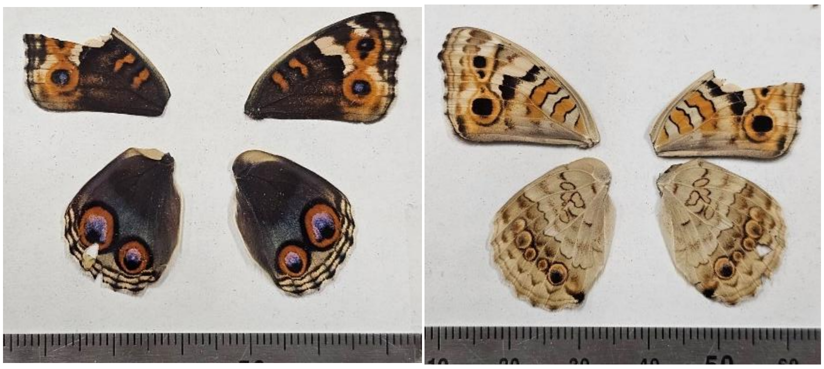

No. 14

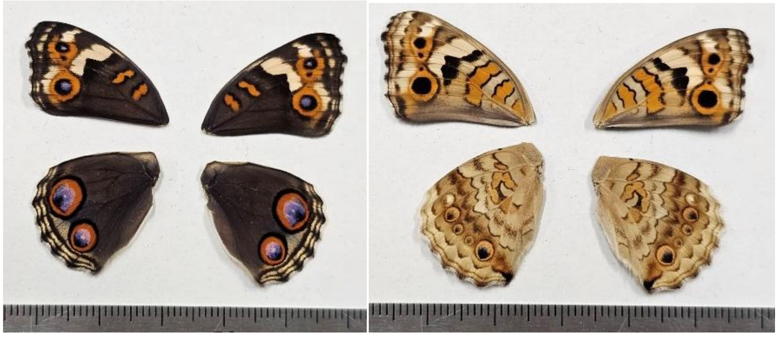

No. 15

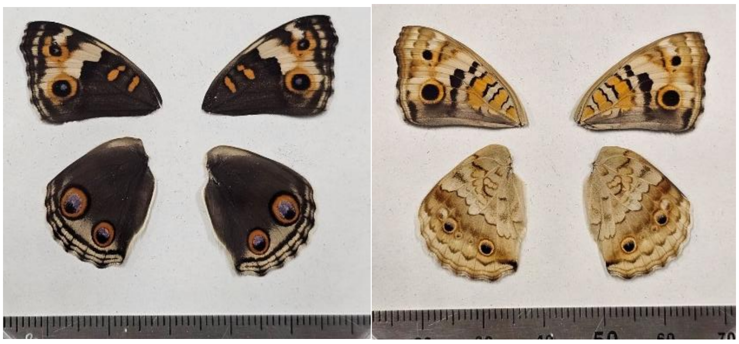

No. 16

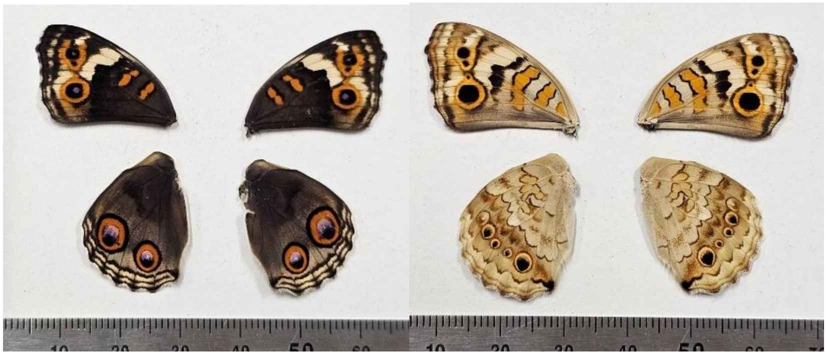

No. 17

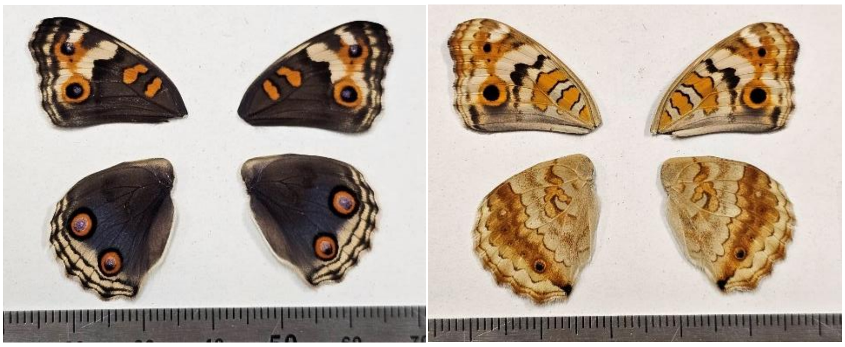

No. 18

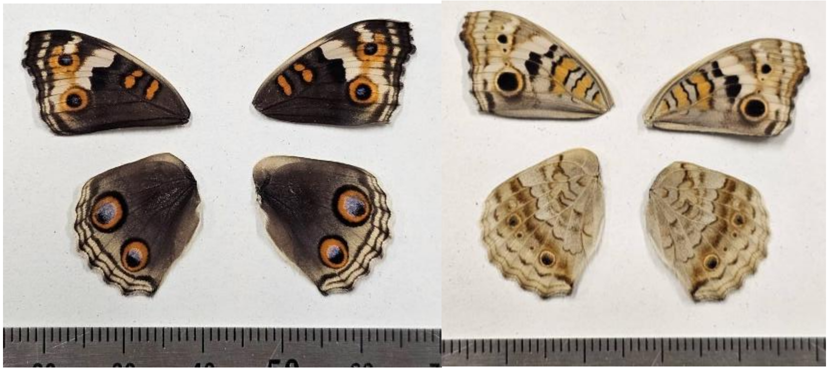

No. 19

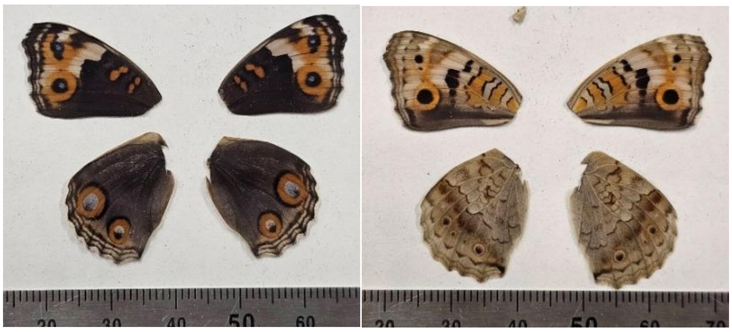

No. 20

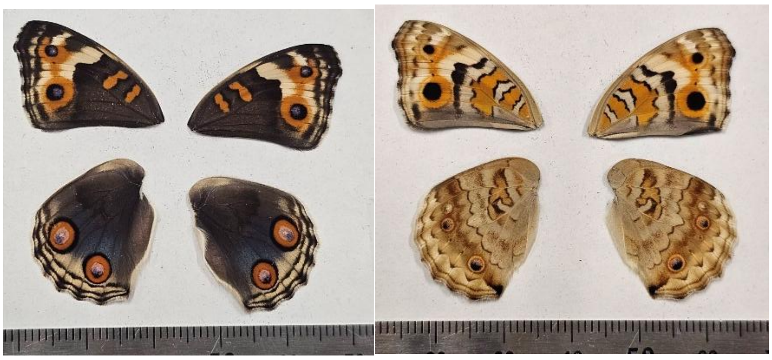

No. 21

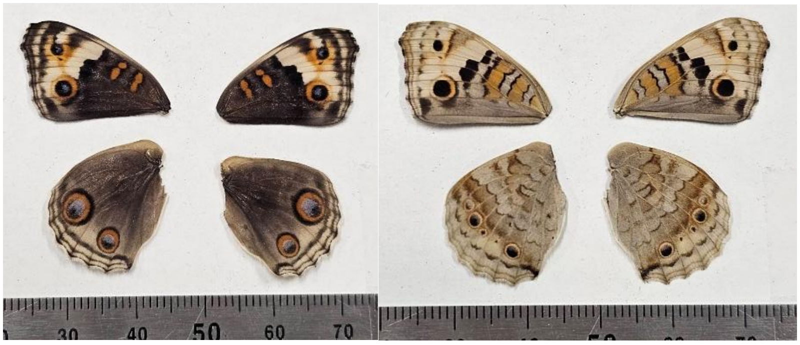

No. 22

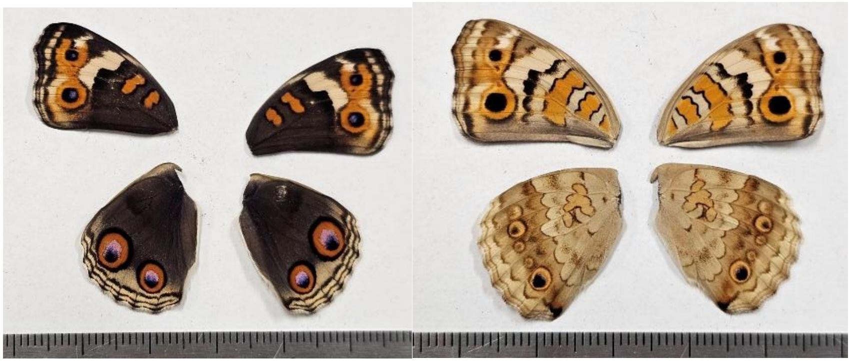

No. 23

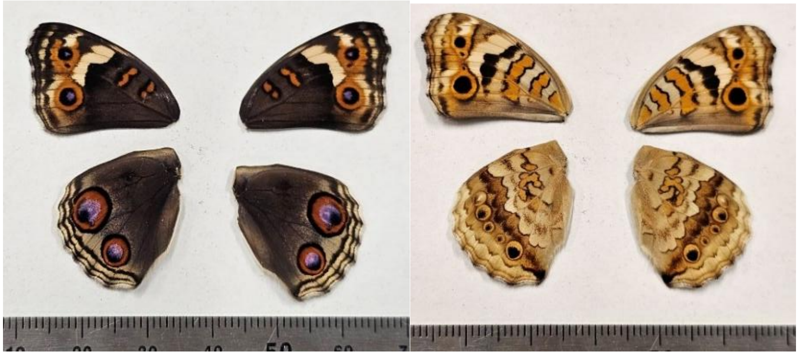

No. 24

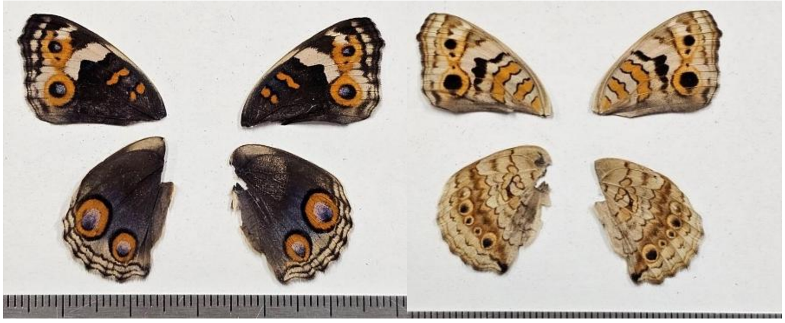

(**b**) DMSO treatment (n = 18).

No. 1

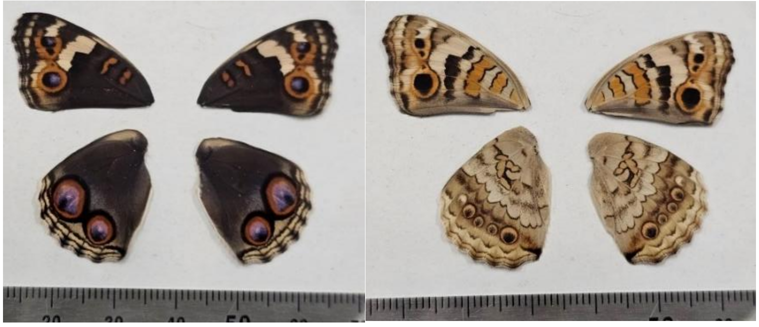

No. 2

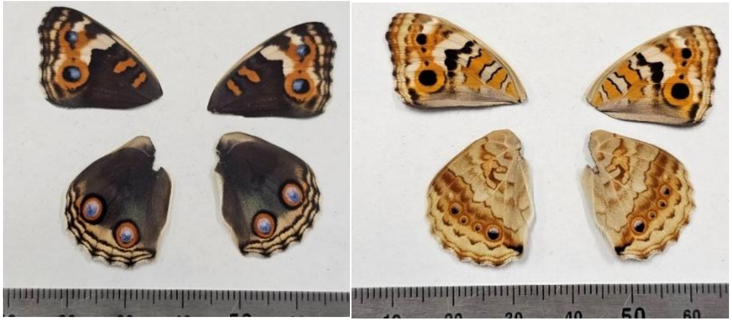

No. 3

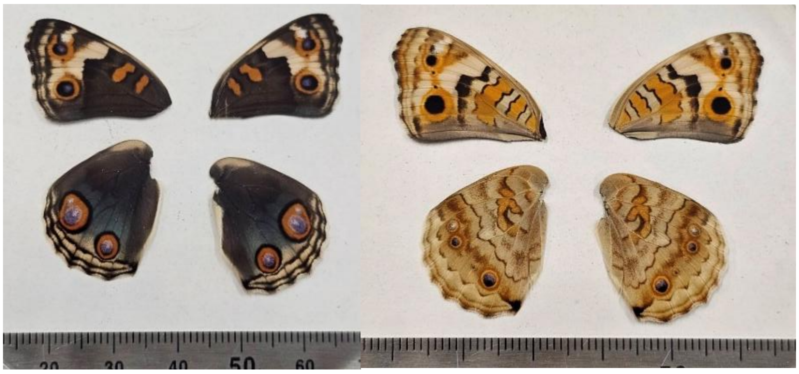

No. 4

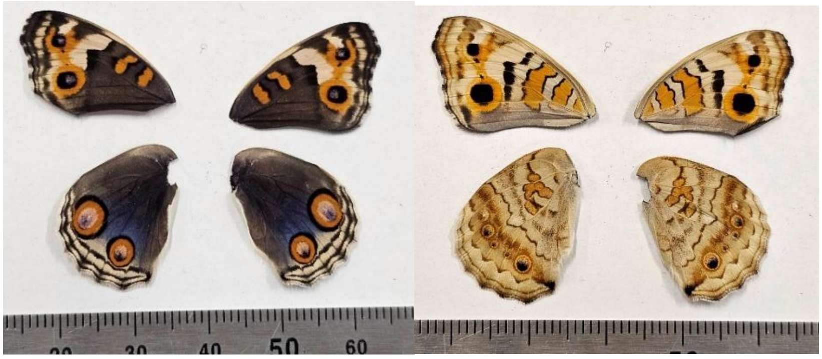

No. 5

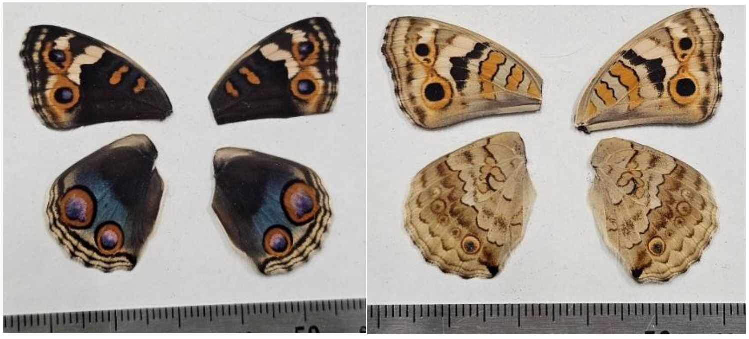

No. 6

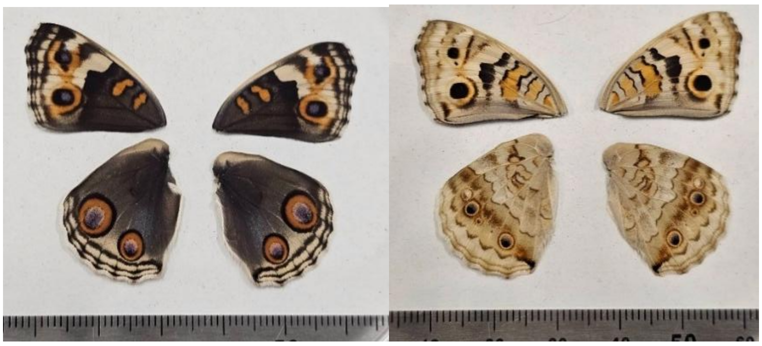

No. 7

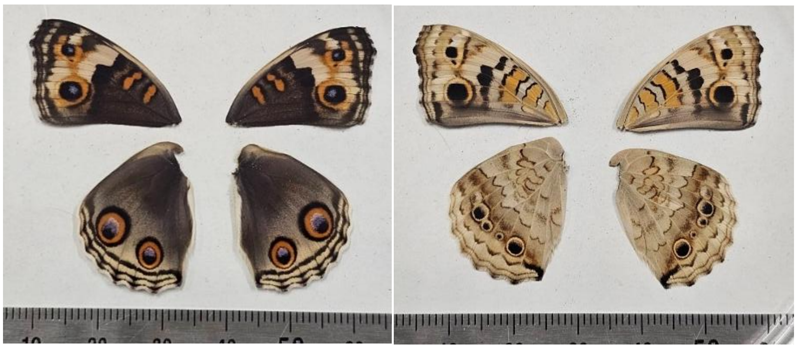

No. 8

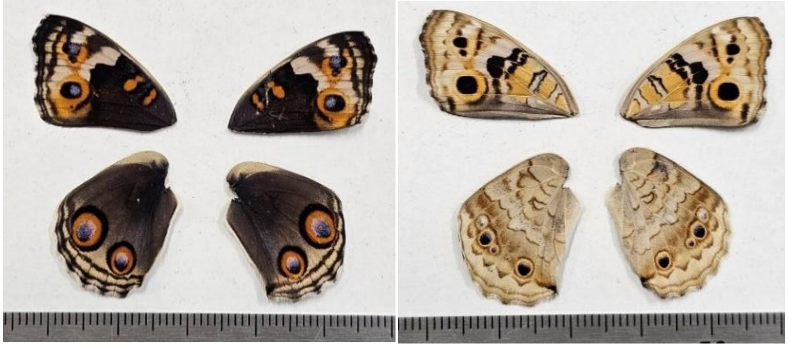

No. 9

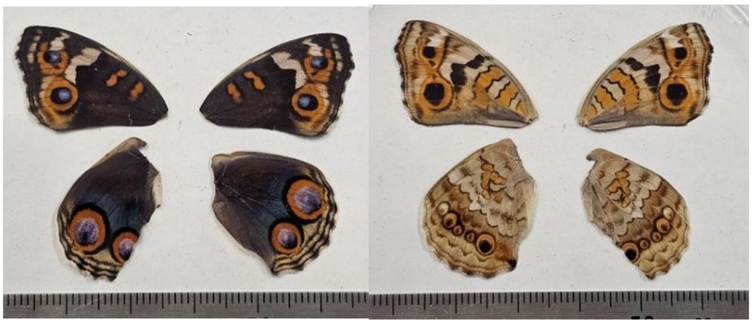

No. 10

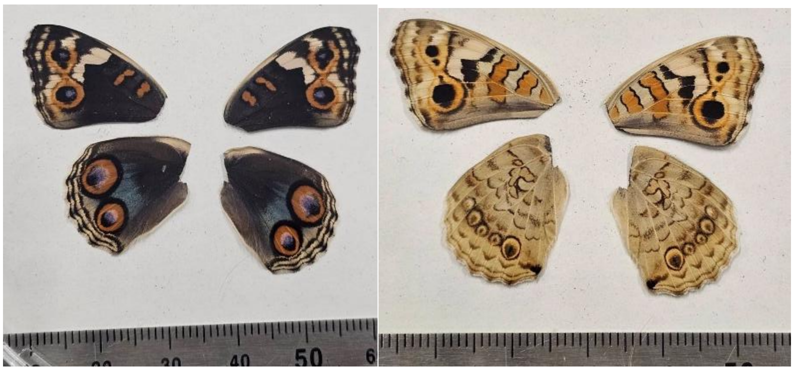

No. 11

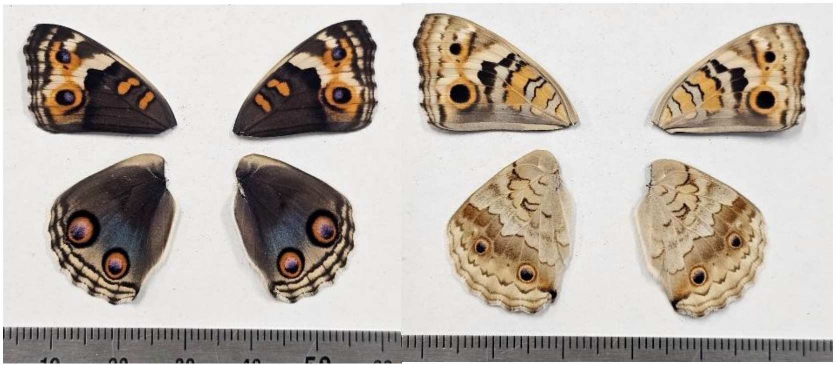

No. 12

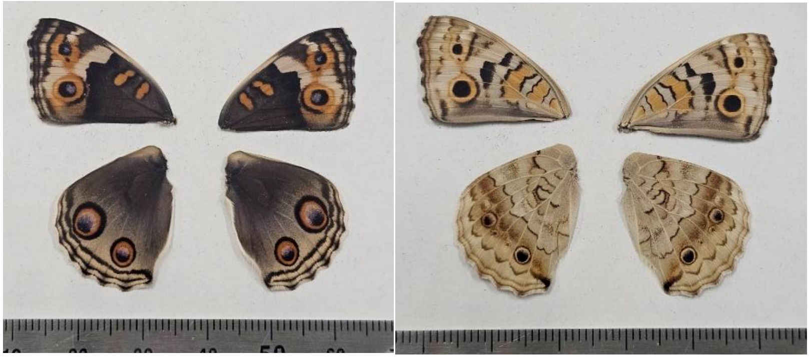

No. 13

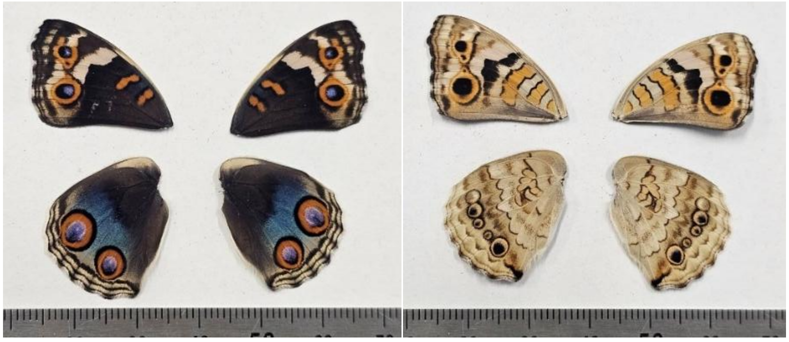

No. 14

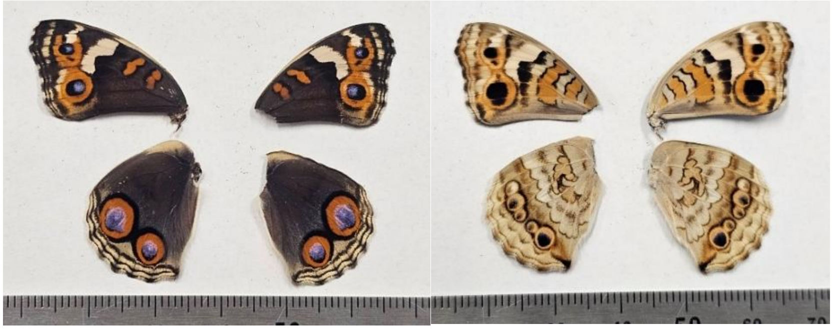

No. 15

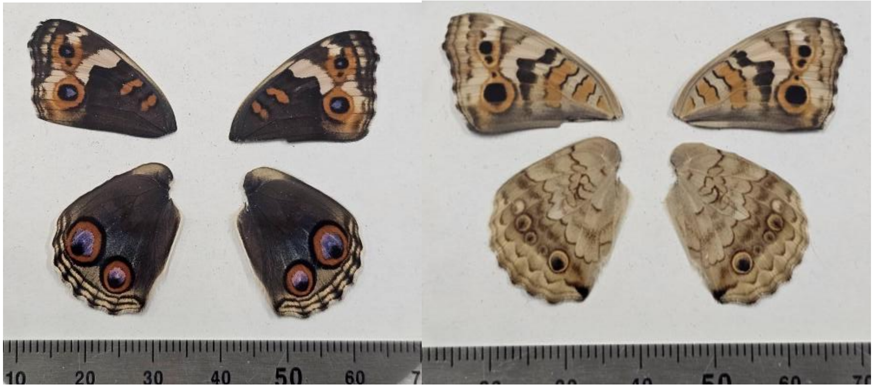

No. 16

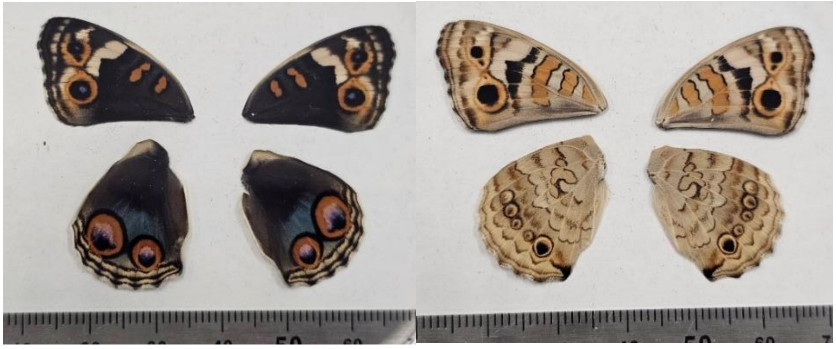

No. 17

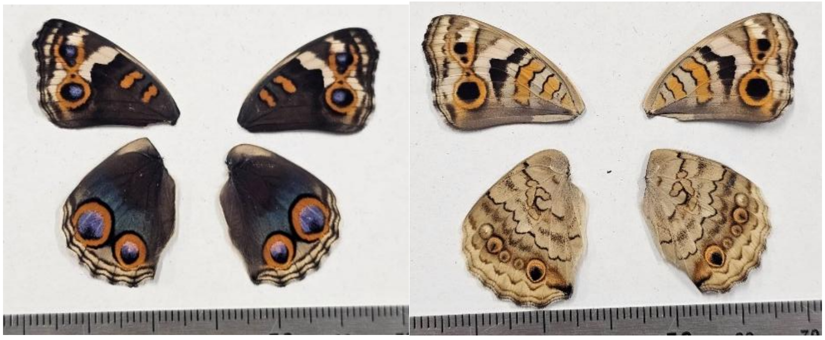

No. 18

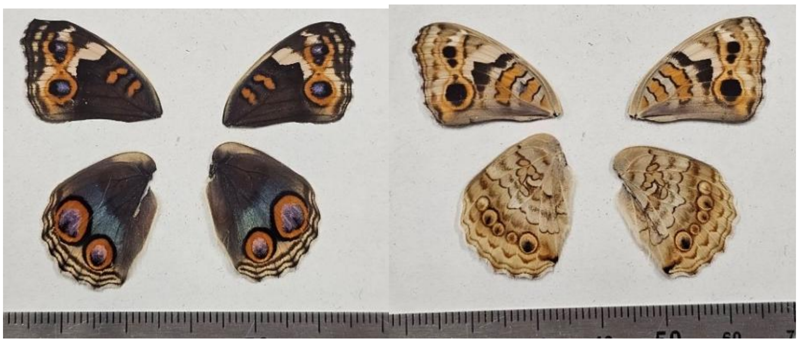

(**c**) AM0902 treatment (*n* = 11).

No. 1

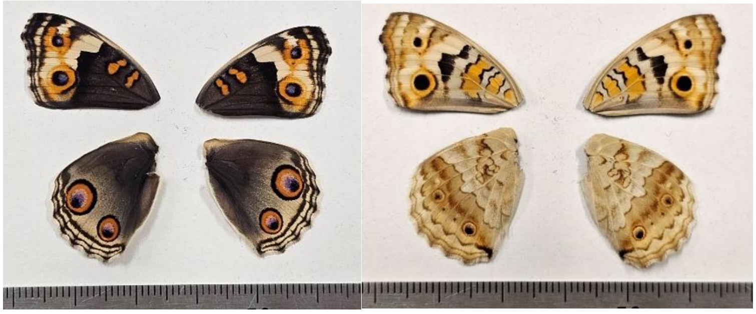

No.2

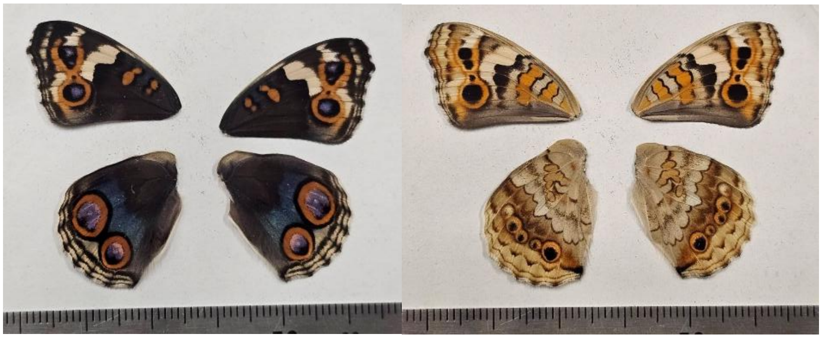

No.3

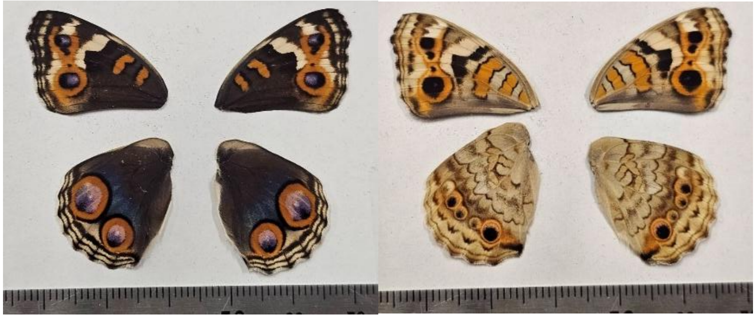

No.4

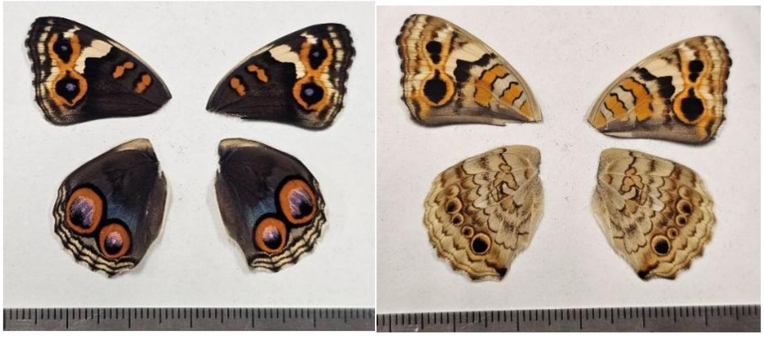

No.5

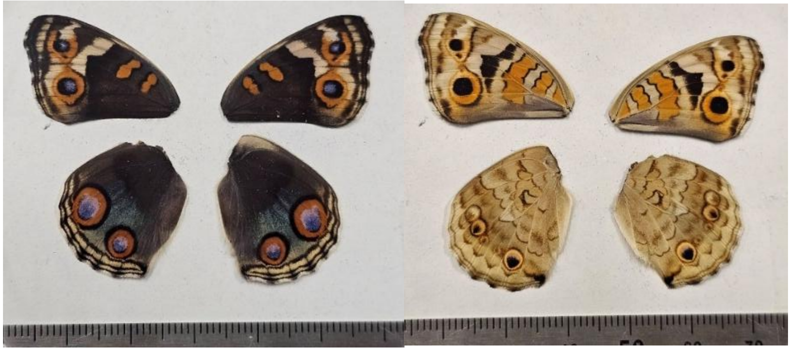

No.6

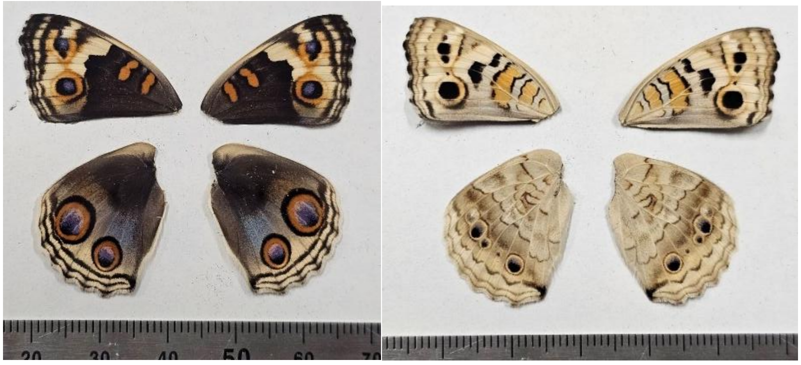

No.7

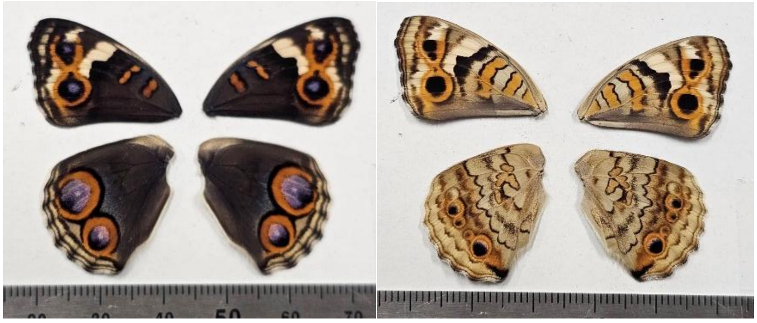

No. 8

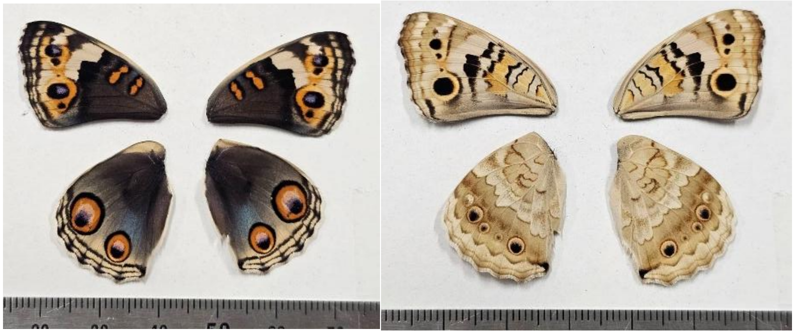

No. 9

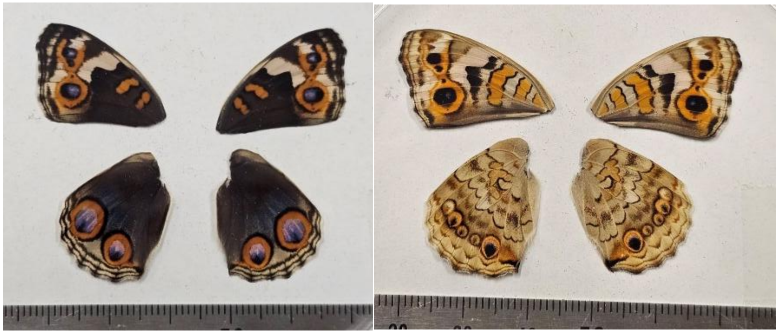

No. 10

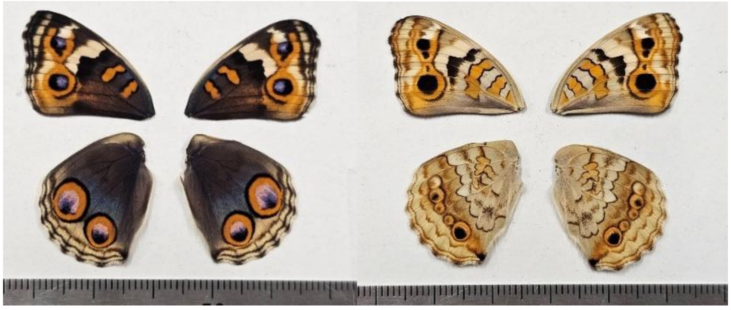

No. 11

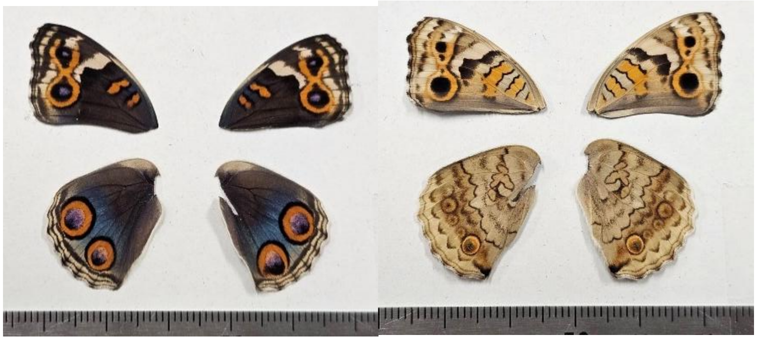

(**d**) AP-18 treatment (n = 9).

No.1

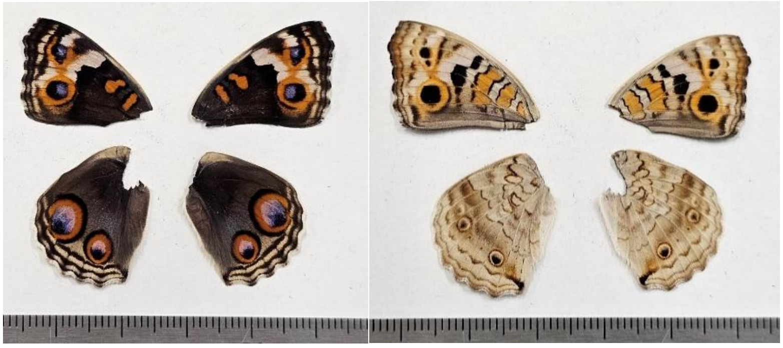

No.2

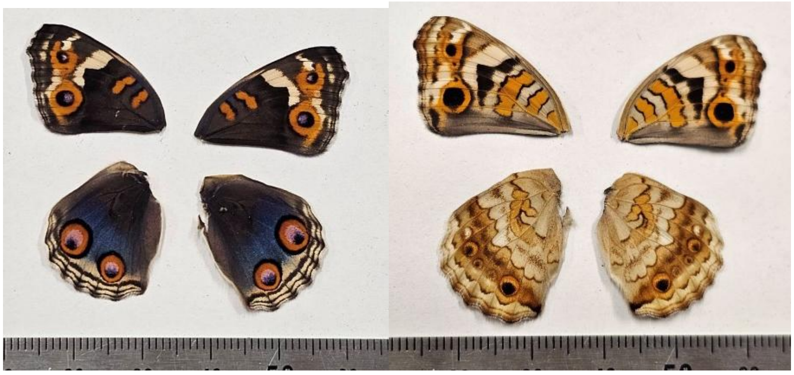

No.3

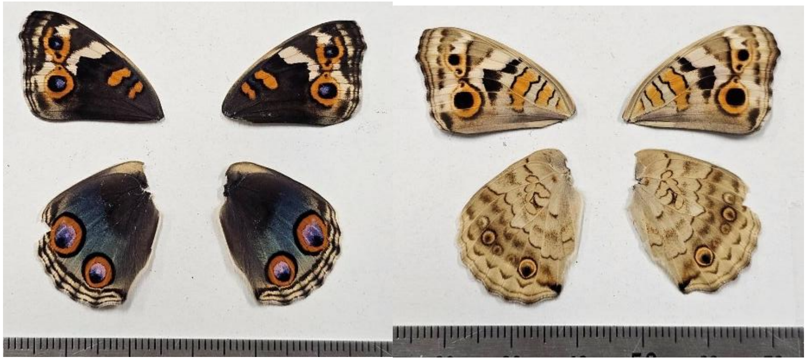

No.4

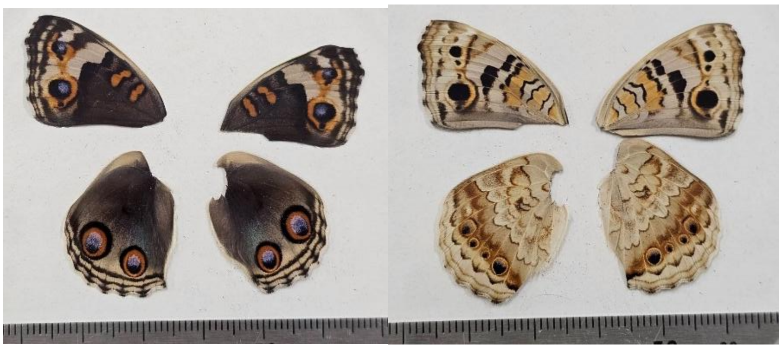

No.5

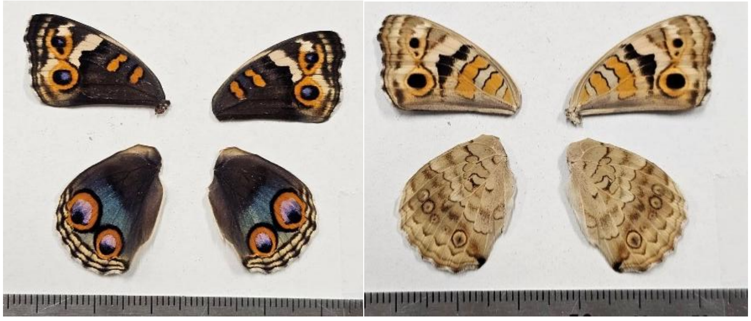

No.6

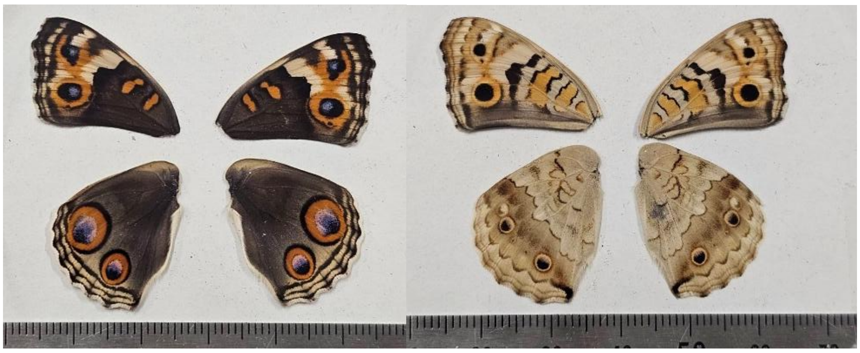

No.7

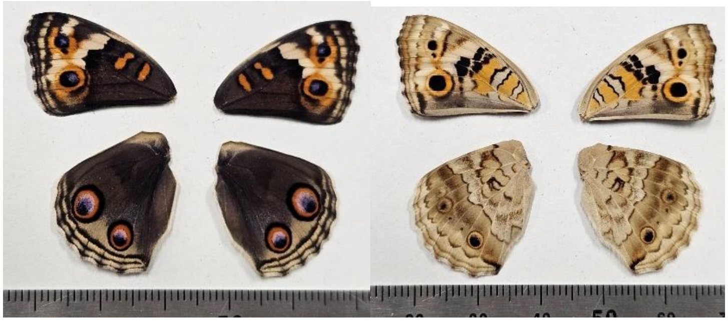

No. 8

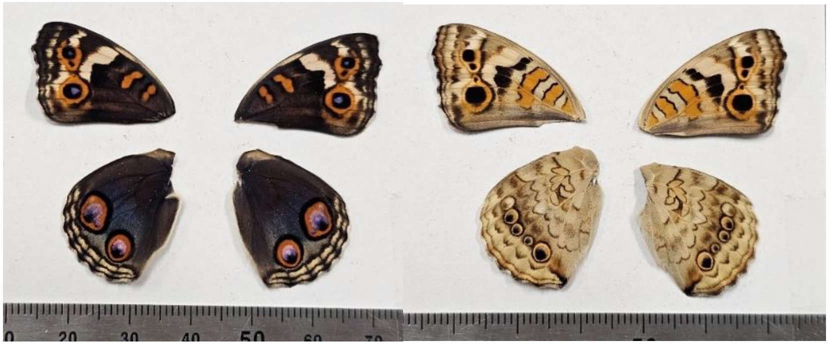

No. 9

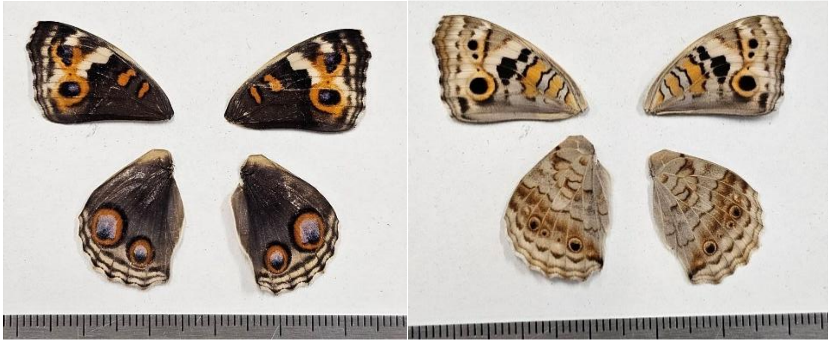

(**e**) Anti-TRPA1-Ex antibody treatment (*n* = 15).

No. 1

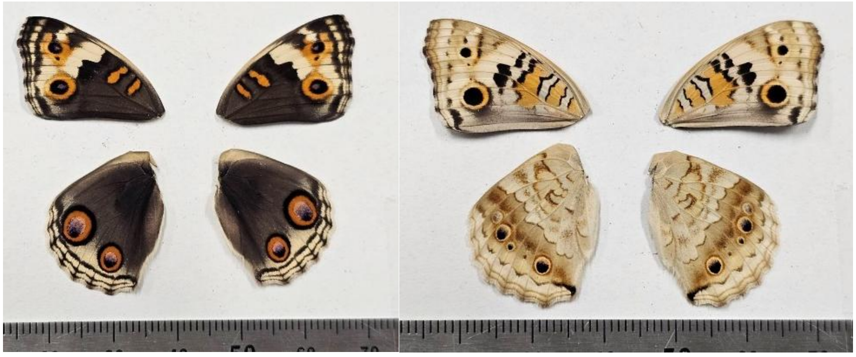

No. 2

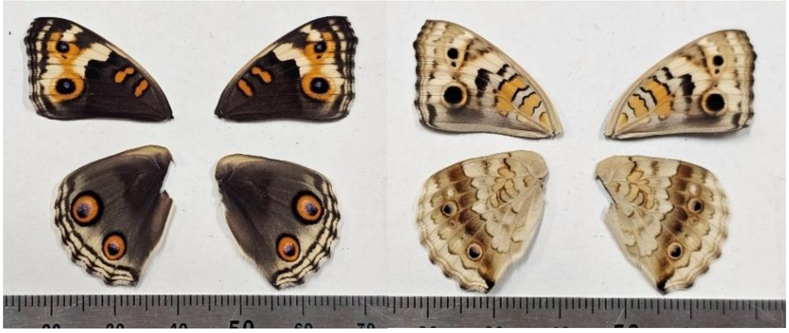

No. 3

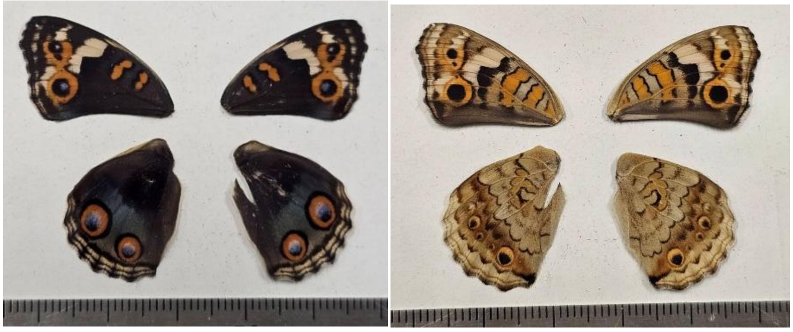

No. 4

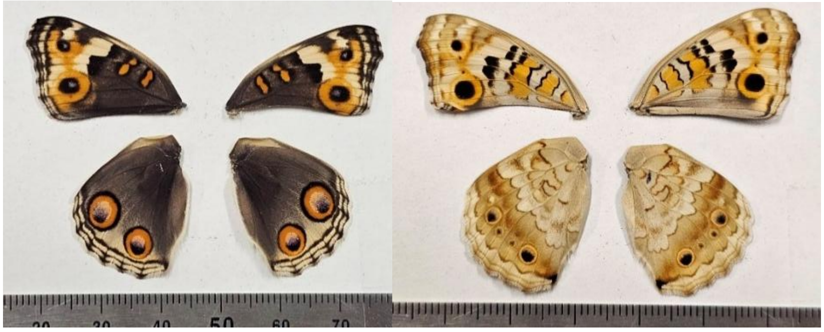

No. 5

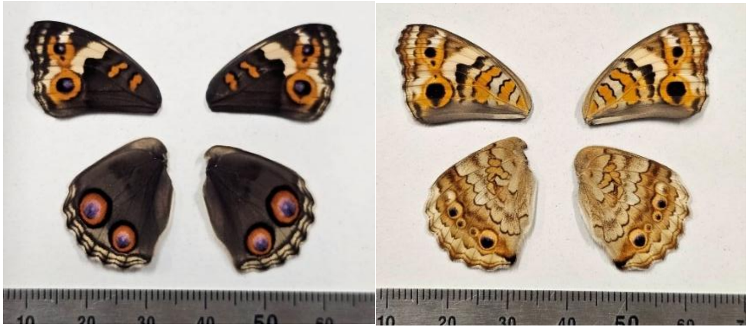

No. 6

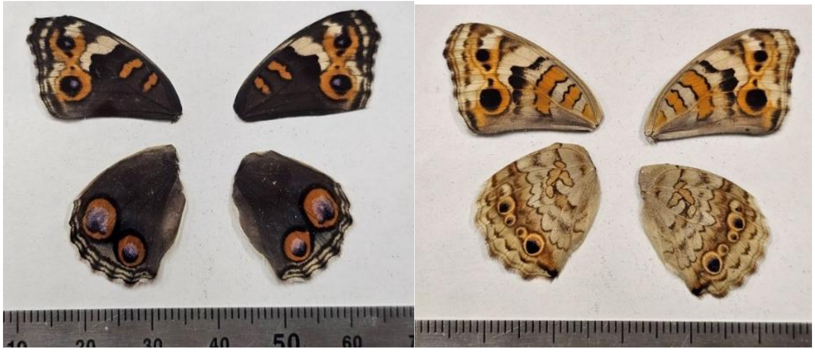

No. 7

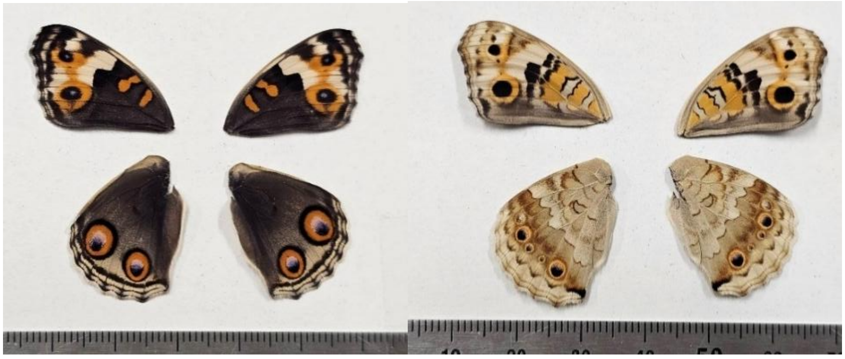

No. 8

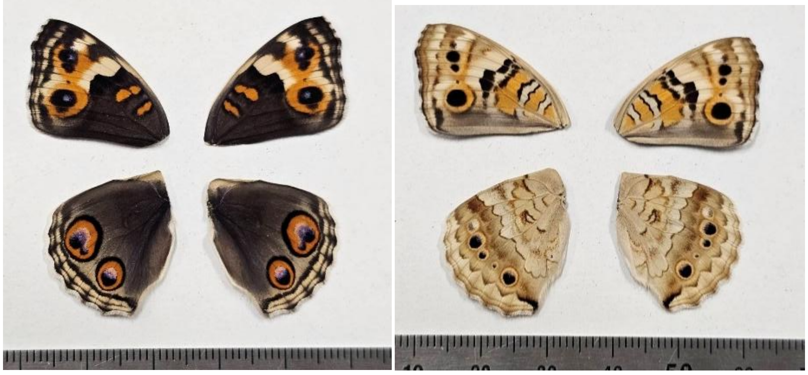

No. 9

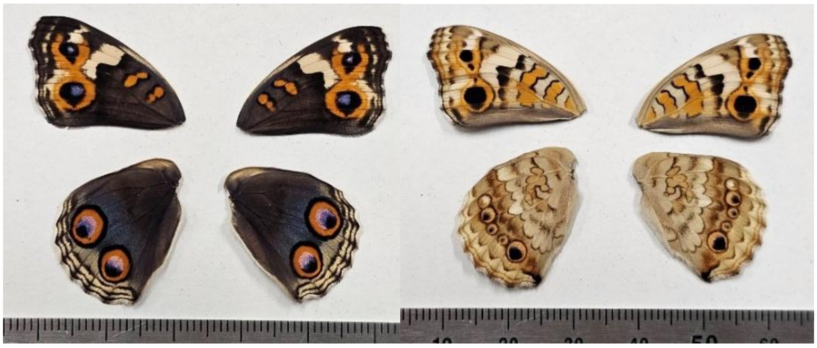

No. 10

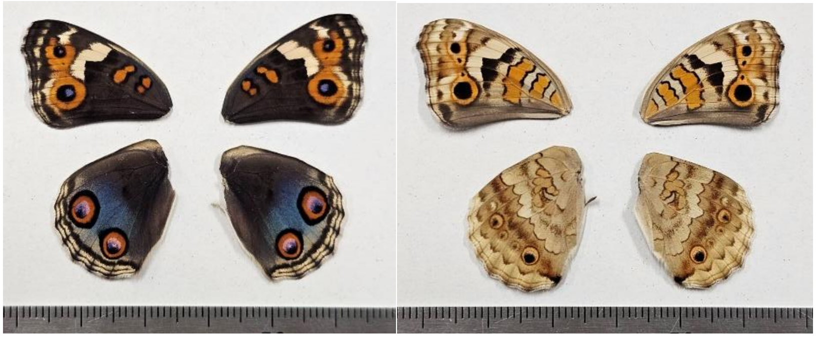

No. 11

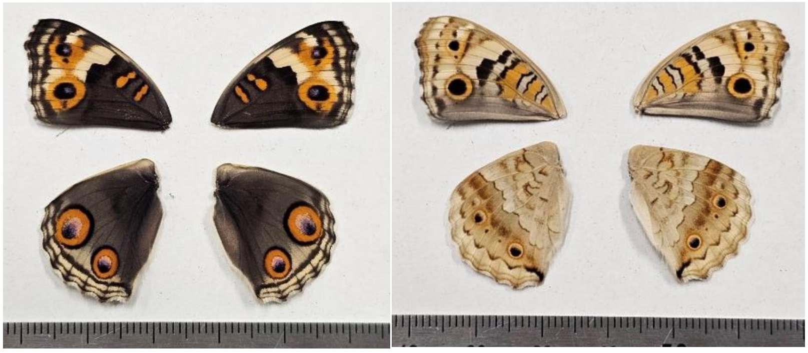

No. 12

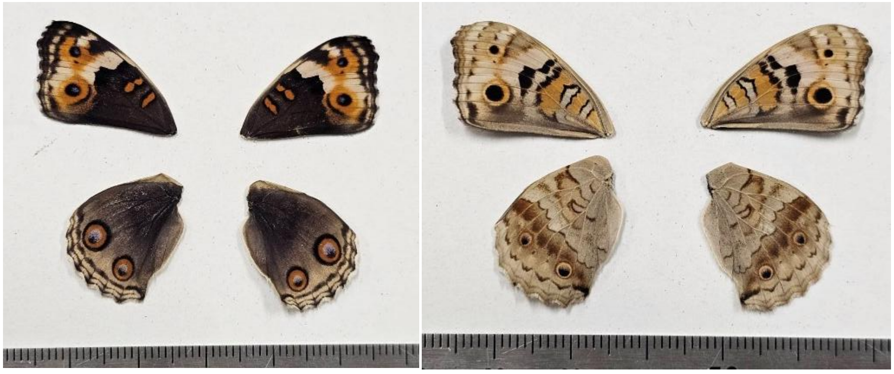

No. 13

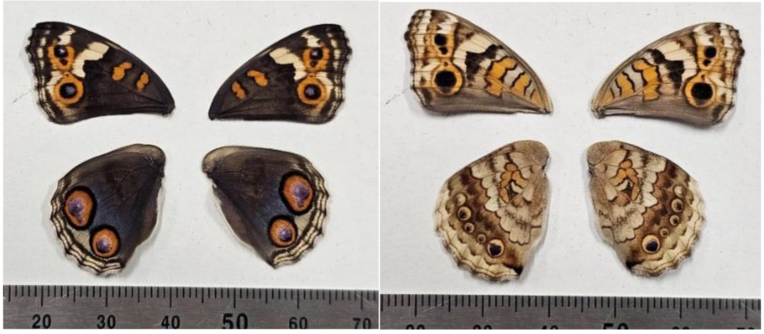

No. 14

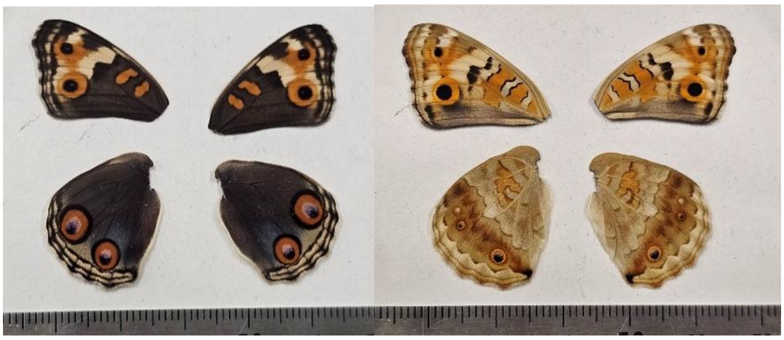

No. 15

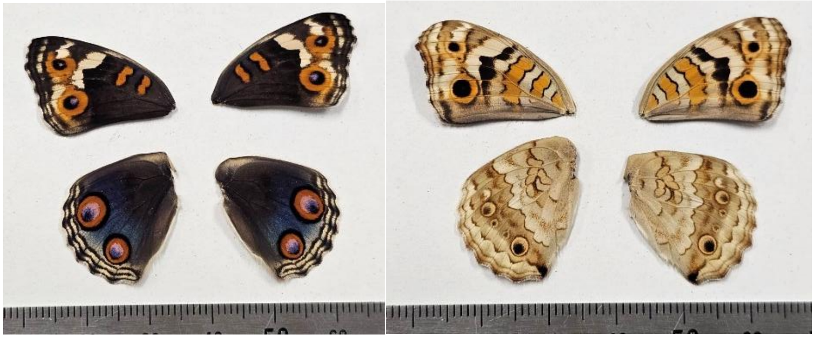

(**f**) Anti-spike P1 antibody (n = 19).

No. 1

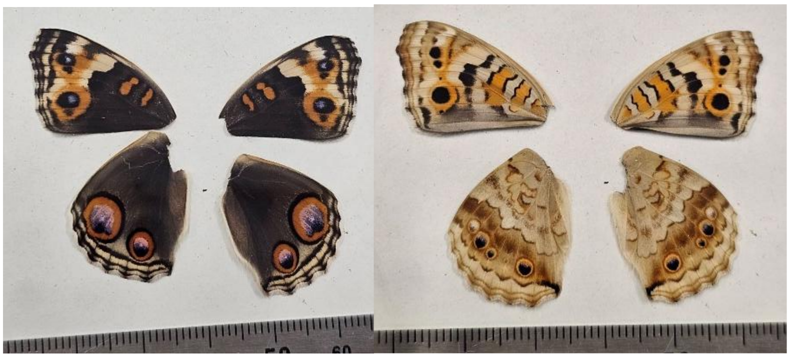

No. 2.

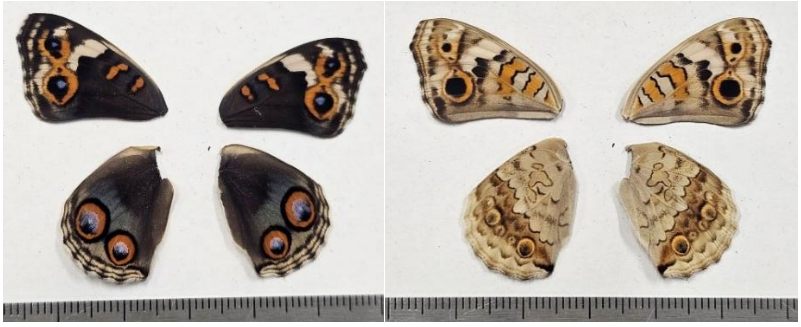

No. 3

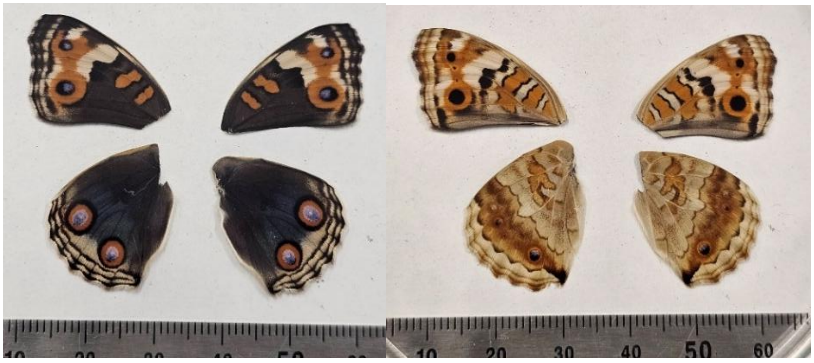

No. 4

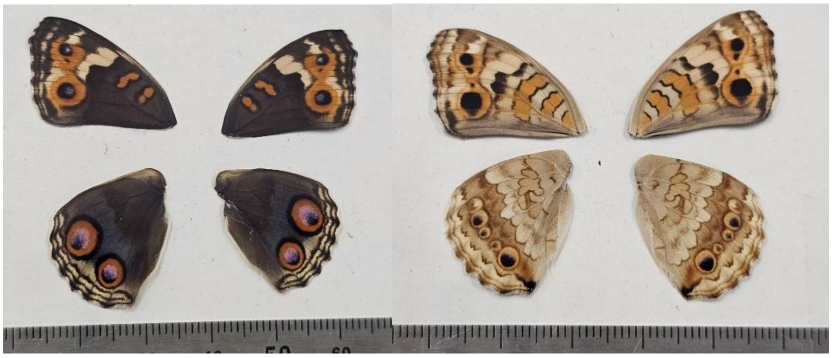

No. 5

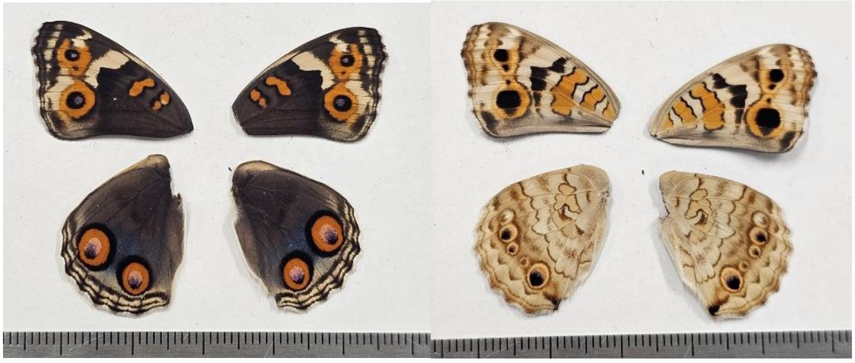

No. 6

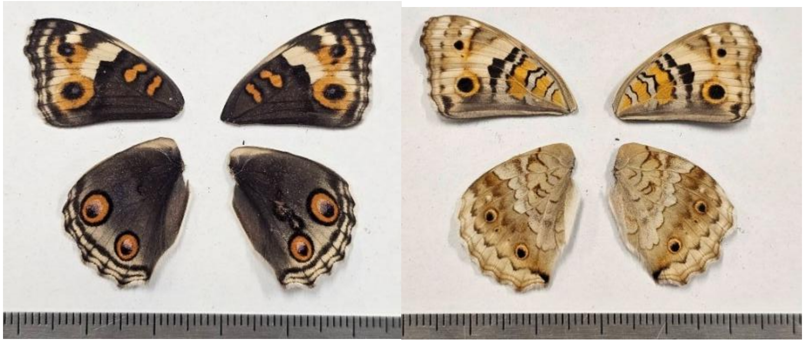

No. 7

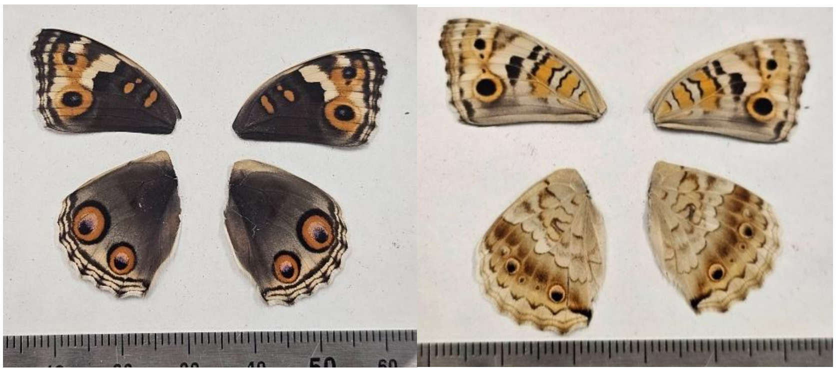

No. 8

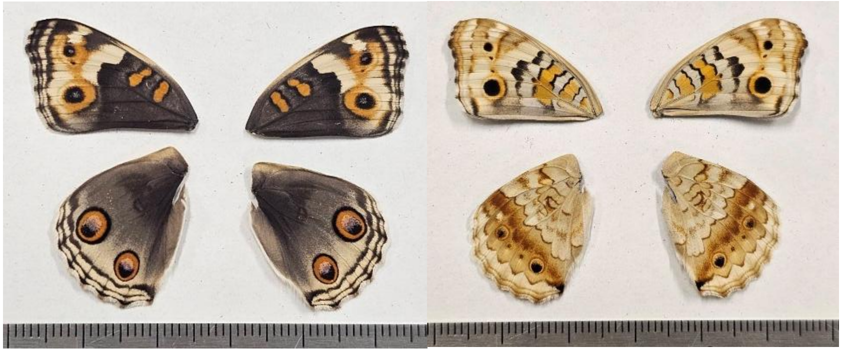

No. 9

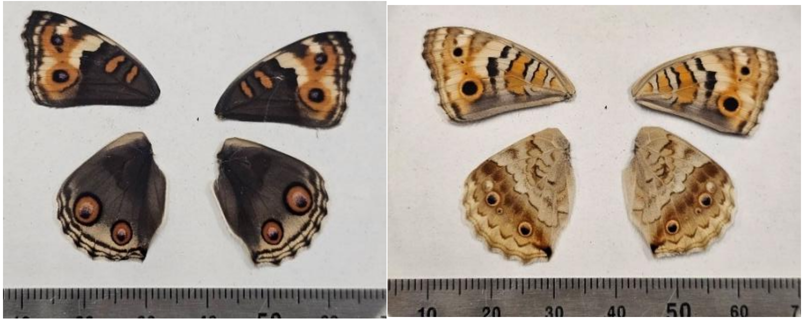

No. 10

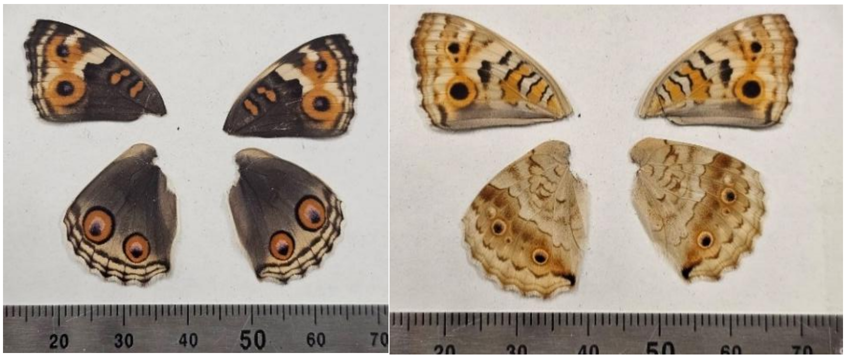

No. 11

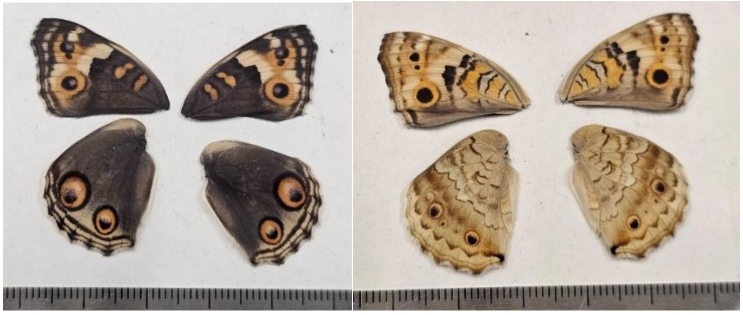

No. 12

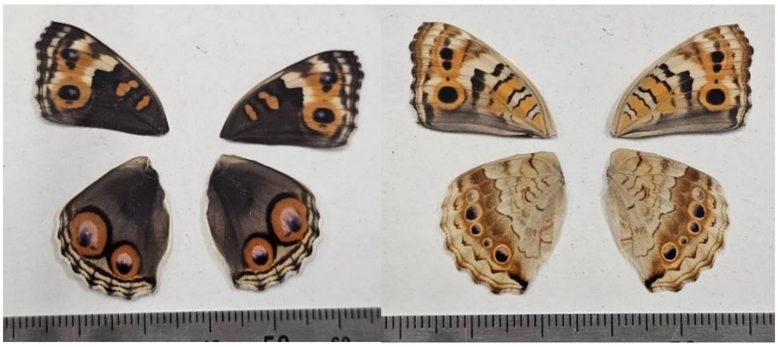

No. 13

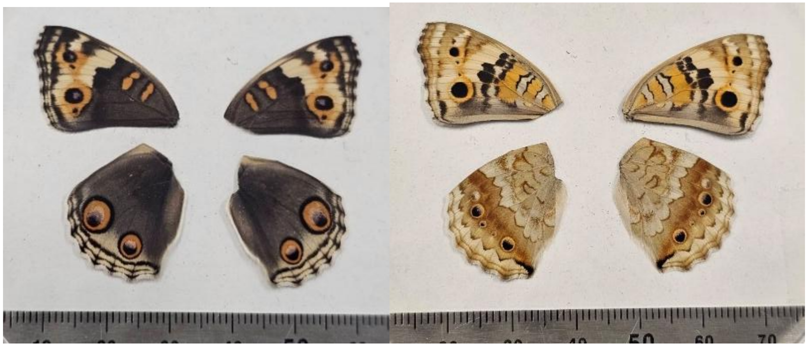

No. 14

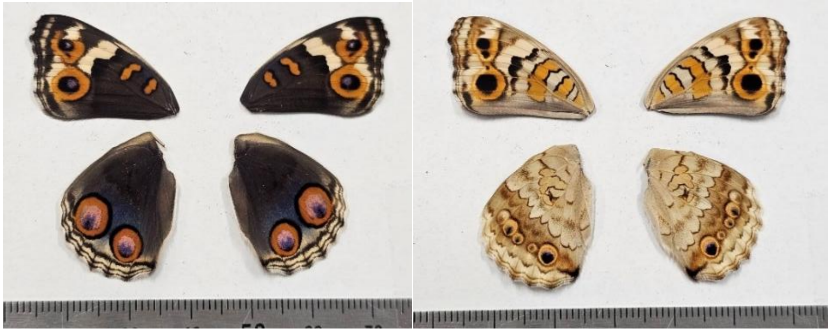

No. 15

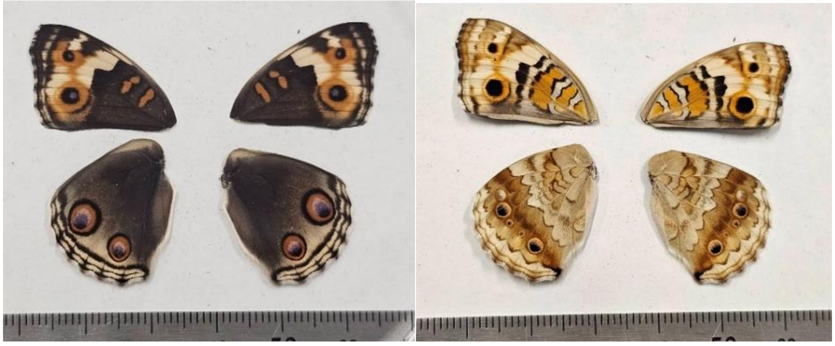

No. 16

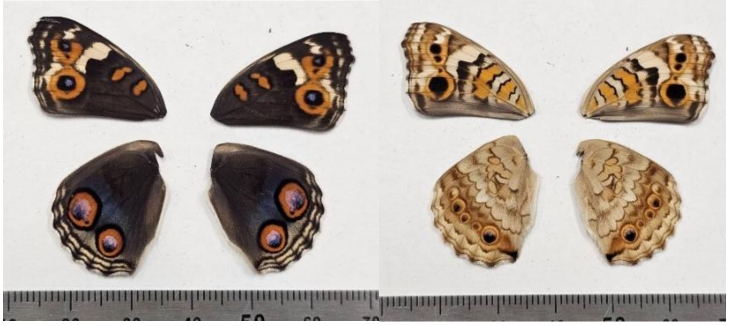

No. 17

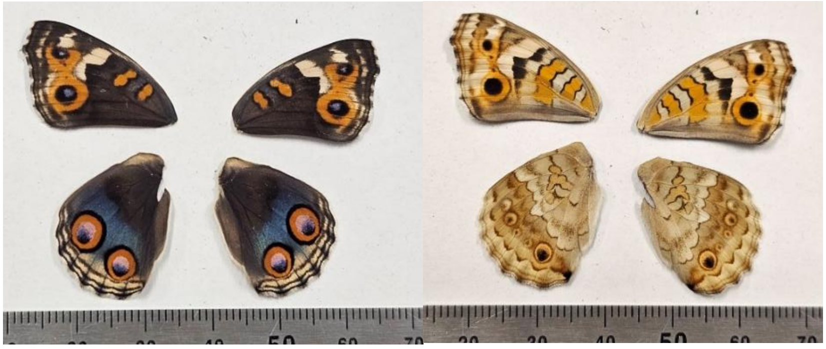

No. 18

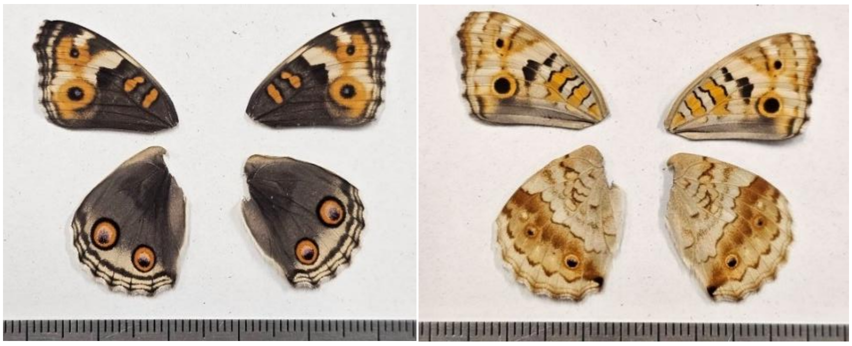

No. 19

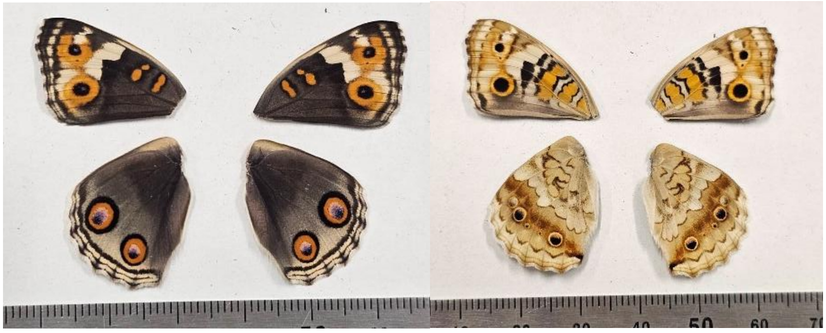

(**g**) Anti-TRPA1-In antibody plus ProteoCarry treatment ($n = 4$).

No. 1

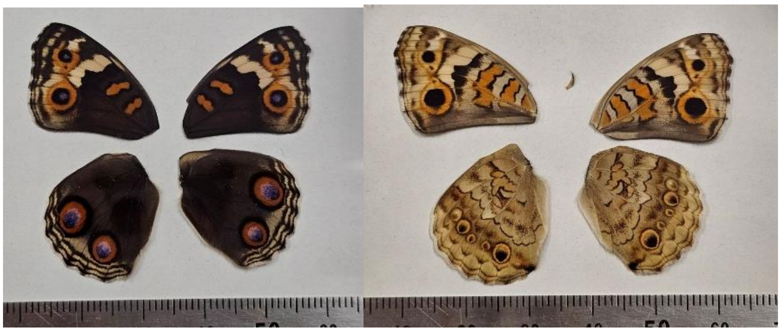

No. 2

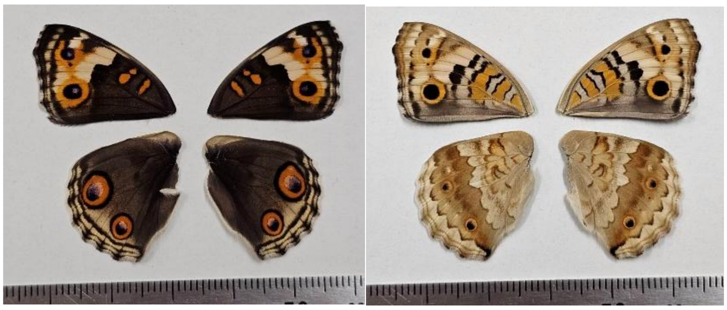

No. 3

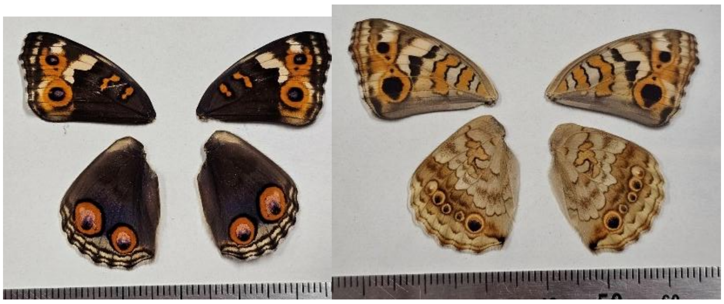

No. 4

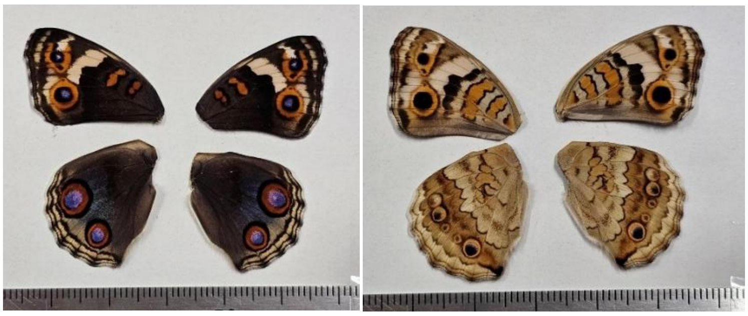

(**h**) Anti-spike P1 antibody plus ProteoCarry treatment (n = 4).

No. 1

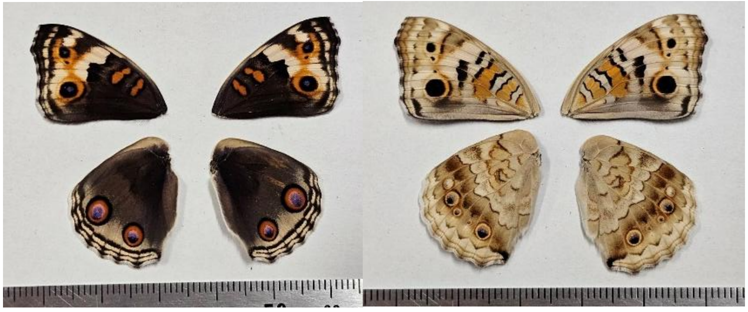

No. 2

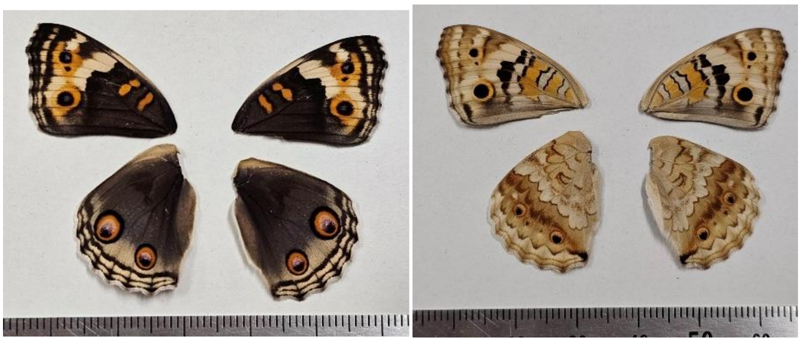

No. 3

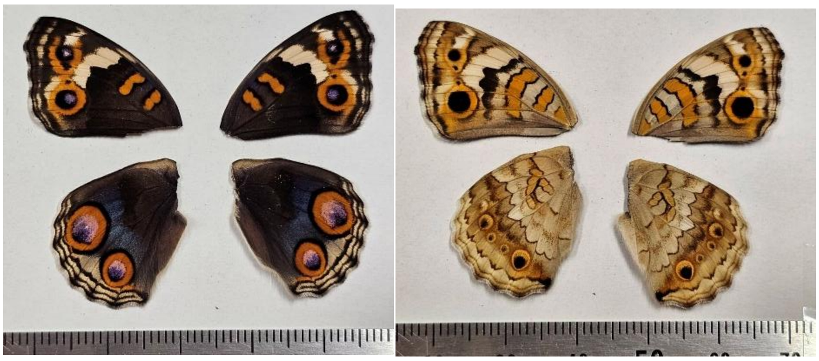

No. 4

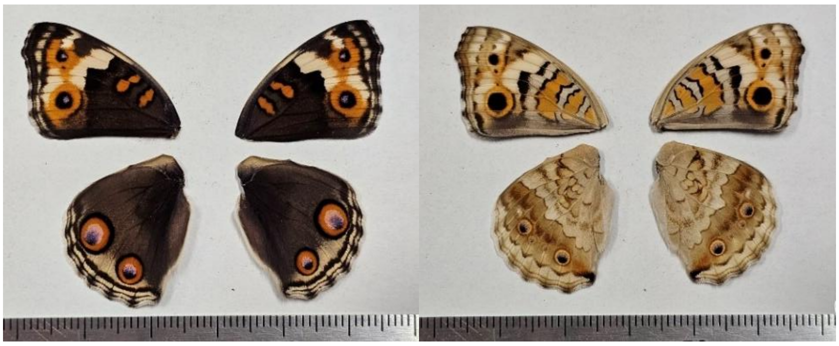

Supplement: Supplementary file 1 [file ijms-27-01420-s001.zip › TRPA1 Supplementary Figure S1.pdf]
